# Structure elucidation of the novel carotenoid gemmatoxanthin from the photosynthetic complex of *Gemmatimonas phototrophica* AP64

**DOI:** 10.1038/s41598-021-95254-6

**Published:** 2021-08-05

**Authors:** Nupur, Marek Kuzma, Jan Hájek, Pavel Hrouzek, Alastair T. Gardiner, Martin Lukeš, Martin Moos, Petr Šimek, Michal Koblížek

**Affiliations:** 1 Centre Algatech, Institute of Microbiology of the Czech Academy of Sciences, 379 81 Třeboň, Czech Republic; 2 Laboratory of Molecular Structure Characterization, Institute of Microbiology of the Czech Academy of Sciences, Vídeňská 1083 Prague, Czech Republic; 3 Faculty of Science, University of South Bohemia, Branišovská 1760 České Budějovice, Czech Republic; 4 Biology Centre of the Czech Academy of Sciences, Branišovská 1160/31, 370 05 České Budějovice, Czech Republic

**Keywords:** Mass spectrometry, Natural product synthesis

## Abstract

*Gemmatimonas phototrophica* AP64 is the first phototrophic representative of the bacterial phylum *Gemmatimonadetes*. The cells contain photosynthetic complexes with bacteriochlorophyll *a* as the main light-harvesting pigment and an unknown carotenoid with a single broad absorption band at 490 nm in methanol. The carotenoid was extracted from isolated photosynthetic complexes, and purified by liquid chromatography. A combination of nuclear magnetic resonance (^1^H NMR, COSY, ^1^H-^13^C HSQC, ^1^H-^13^C HMBC, *J*-resolved, and ROESY), high-resolution mass spectroscopy, Fourier-transformed infra-red, and Raman spectroscopy was used to determine its chemical structure. The novel linear carotenoid, that we have named gemmatoxanthin, contains 11 conjugated double bonds and is further substituted by methoxy, carboxyl and aldehyde groups. Its IUPAC-IUBMB semi-systematic name is 1′-Methoxy-19′-oxo-3′,4′-didehydro-7,8,1′,2′-tetrahydro- Ψ, Ψ carotene-16-oic acid. To our best knowledge, the presence of the carboxyl, methoxy and aldehyde groups on a linear C40 carotenoid backbone is reported here for the first time.

## Introduction

Photosynthesis is an ancient process that probably evolved more than 3 billion years ago^1^. It is believed that the earliest phototrophic organisms were anoxygenic (not producing oxygen) species^2^, which throughout evolution diverged into a number of bacterial phyla. The latest phylum from which anoxygenic phototrophic species have been isolated is *Gemmatimonadetes.* The phylum *Gemmatimonadetes* was formally established in 2003, with *Gemmatimonas* (*G*.) *aurantiaca* as the type species. Even though there exists only a handful of cultured strains, Gemmatimonadetes are known to be relatively common organisms in many natural habitats, such as soils, sediments and freshwaters^3,4^. To date, two phototrophic species have been described in the genus *Gemmatimonas*: *G. phototrophica* AP64, originating from a freshwater lake in the Gobi Desert^5^, and *G. groenlandica* TET16 isolated from a freshwater stream in Greenland^6^.

So far most of the research focused on phototrophy in *Gemmatimonadetes* has been conducted with the facultatively photoheterotrophic strain, *G. phototrophica* AP64. It requires a supply of organic carbon substrates for metabolism and growth. However, light energy through photosynthesis can supplement the metabolic requirements of the cell and improve the organic carbon growth efficiency^7^. The photosynthesis genes in *G. phototrophica* are organized in a 42.3-kb photosynthesis gene cluster (PGC), the organization of which closely resembles that of Proteobacteria and indicates that phototrophic Gemmatimonadetes received their photosynthesis genes horizontally from Proteobacteria^8^.

*G. phototrophica* contains bacteriochlorophyll (BChl) *a* as the main light-harvesting pigment, however, the cells are strongly red-pink pigmented due to presence of more than 10 different, as yet mostly unknown, carotenoids^8^. Some polar carotenoids present in *G. phototrophica* have been previously identified in the heterotrophic species *G. aurantiaca* T-27. These carotenoids were identified as oscillol 2,2′-dirhamnosides^8,9^ and are unique to this bacterial genus. These carotenoids presumably have a photoprotective role to prevent the formation of reactive oxygen species (ROS)^10^ and may also stabilize bacterial membranes.

Recently, the photosynthetic complex of *G*. *phototrophica* AP64 has been purified and characterized^11^. The complex consists of a bacterial type 2 reaction center (RC) surrounded by two concentric rings of light-harvesting antenna. This unique organization ensures that the complex is highly efficient at harvesting photons and directing them to the RC^11^. The purified complex has good structural stability and contains BChl molecules with phytol or geranyl–geranyl sidechains. The complex also contains carotenoid, however, not an oscillol-rhamnoside derivative, rather a purple colored, as yet unknown carotenoid.

This study aimed to purify and spectroscopically characterize this unknown carotenoid to determine its chemical structure and propose a tentative biosynthetic pathway.

## Results

### Identification of the unknown carotenoid from purified photosynthetic complex

**Figure 1:**
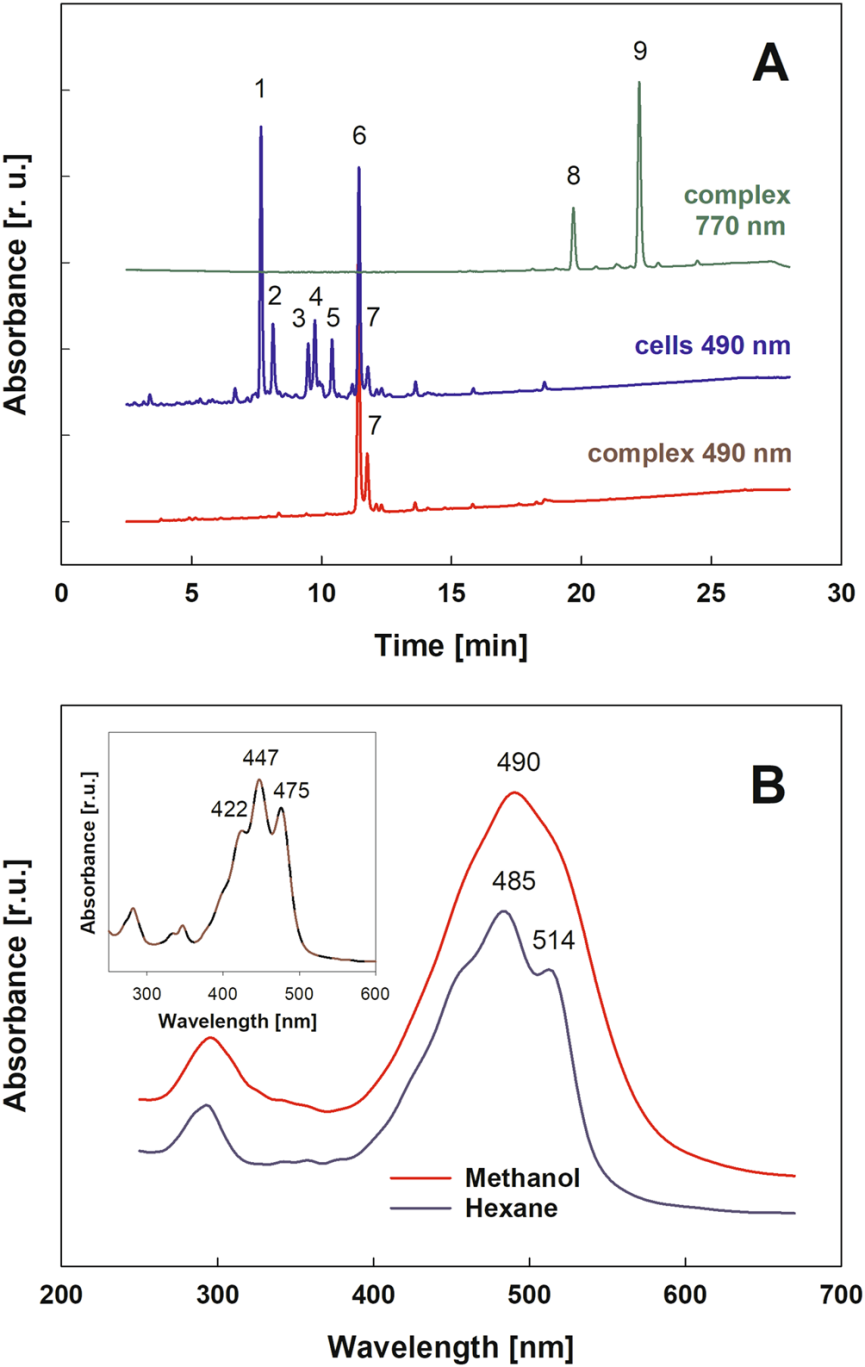
(**A**) HPLC elution profile of *Gemmatimonas phototrophica* AP64 whole-cell extract (green and blue trace) and of the isolated photosystems (red trace) at 490 nm for carotenoids and 770 nm for bacteriochlorophyll, respectively. Identified peaks: 1 and 2, putative (2*S*,2′*S*)-oscillol 2,2′-di-(α-L-rhamnoside); 3–5, unknown carotenoids; 6–7, gemmatoxanthin; 8, BChl *a*_GG_; 9, BChl *a*_P_. (**B**) Absorption spectrum of purified carotenoid corresponding to peak 6 in methanol (red) and hexane (blue). The insert depicts the absorption spectrum after NaBH_4_ reduction in methanol (brown). r.u. – relative units.

*G. phototrophica* AP64 cells contain seven major and several minor carotenoids (Fig. 1). The main carotenoids are (peak 1–2) are oscillol 2,2′-dirhamnosides (Fig. 1A). These polar carotenoids were identified earlier in *G. aurantiaca*^9^. During purification of the PS complex, the majority of carotenoids were removed through sucrose density gradient centrifugation, ion-exchange chromatography and gel filtration (Fig. S1). The purified photosynthetic complex was subjected to solvent extraction and HPLC so that an unknown carotenoid eluted at 11.4 min with a single absorption maxima of 490 nm (Fig. 1B).

This unknown carotenoid was isolated by preparative HPLC. The HRMS analysis of the purified carotenoid provided molecular ions at *m*/*z* 613.4268 [M+H]^+^ and 611.4108 [M−H]^–^ (calculated for C_41_H_57_O_4_, − 2.7 ppm and C_41_H_55_O_4_^−^, 0.305 ppm) in positive and negative modes, respectively. This led to an unambiguous determination of the summary molecular neutral formula C_41_H_56_O_4_.

The absorption spectrum of the unknown carotenoid in methanol has a single broad band with λ_max_ at 490 nm. The solubility of the pure carotenoid in hexane was very limited. In contrast to the methanol spectrum, the spectrum in hexane has the three characteristic carotenoid transition peaks at (455), 485 and 514 nm (Fig. 1B). Since such spectral behavior is typical for carotenoids containing a carbonyl group^12^, we tested its presence through chemical reduction using NaBH_4_. As expected, the spectrum of the reduced compound in methanol revealed the three peaks with absorption maxima at 422, 447, 475 nm and its fine structure (% III/II ratio) is 37% (Fig. 1B). It is the ratio of the absorption peak height from the trough between the longest-wavelength peaks (III) to the middle wavelength peak (II). The molecular mass of the reduced compound increased to 2.0165 Da. This indicated the presence of a single carbonyl group connected to the system of conjugated double bonds.

Since the initial spectroscopic studies and MS data of an unknown purified carotenoid indicated that it may represent a novel chemical structure, we named this carotenoid gemmatoxanthin and conducted its full structural characterization.

### Nuclear magnetic resonance

Due to the limited amount of gemmatoxanthin it was not possible to acquire a ^13^C NMR spectrum directly. Therefore, *G. phototrophica* cells were grown on the ^13^C-labeled substrate and the experiment was conducted with ^13^C enriched gemmatoxanthin. The information about carbon chemical shifts had to be obtained indirectly by ^1^H-^13^C HSQC and ^1^H-^13^C HMBC correlation experiments (Table 1, Fig. S2-S10). The ^1^H NMR spectra are provided in the supplementary Fig. S11-S14.

**Table 1:** The ^1^H and ^13^C NMR data of gemmatoxanthin (**1**) (700.13 MHz for ^1^H, 176.05 for ^13^C, DMSO-*d*_*6*_, 303.1 K).

Position #	δ_C_	mult	δ_H_	m	*J*_HH_ [Hz]
1—C	n.d				
2—CH	n.d		6.499	br s	
3—CH_2_	26.7	t	2.182	m	–
4—CH_2_	38.0	t	2.033	m	–
5—C	134.2	s	–		
6—CH	123.9	d	5.126	m	–
7—CH_2_	26.0	t	2.107	m	–
8—CH_2_	39.6	t	2.099	m	–
9—C	140.0	s	–		
10—CH	125.7	d	5.970	d	10.9
11—CH	126.0	d	6.570	dd	10.9, 15.1
12—CH	134.9	d	6.281	d	15.1
13—C	138.4	s	–		
14—CH	131.2	d	6.318	d	11.8
15—CH	133.6	d	6.898	m	–
16—C	169.5	s	–		
17—CH_3_	12.6	q	1.697	s	–
18—CH_3_	15.7	q	1.591	s	–
19—CH_3_	16.7	q	1.802	s	–
20—CH_3_	12.6	q	1.952	s	–
1′—C	74.3	s	–		
2′—CH_2_	43.1	t	2.292	d	7.4
3′—CH	126.9	d	5.771	dt	7.4, 15.5
4′—CH	136.6	d	6.186	d	15.5
5′—C	136.7	s	–		
6′—CH	131.0	d	6.219	d	11.4
7′—CH	130.1	d	7.675	dd	11.4, 15.2
8′—CH	123.0	d	6.752	d	15.2
9′—C	133.0	s	–		
10′—CH	148.9	d	7.055	d	11.5
11′—CH	122.8	d	7.013	dd	11.5, 13.9
12′—CH	147.1	d	6.943	d	13.9
13′—C	135.4	s	–		
14′—CH	138.3	d	6.627	d	11.6
15′—CH	129.3	d	6.743	m	–
16′, 17′—CH_3_	24.7	q	1.091	s	–
18′—CH_3_	12.8	q	1.884	s	–
19′—COH	194.2	d	9.539	d	2.1
20′—CH_3_	12.4	q	2.014	s	–
21′—OMe	48.6	q	3.114	s	–

s: singlet, d: doublet, t: triplet, q: quartet, m: multiplet.

**Figure 2:**
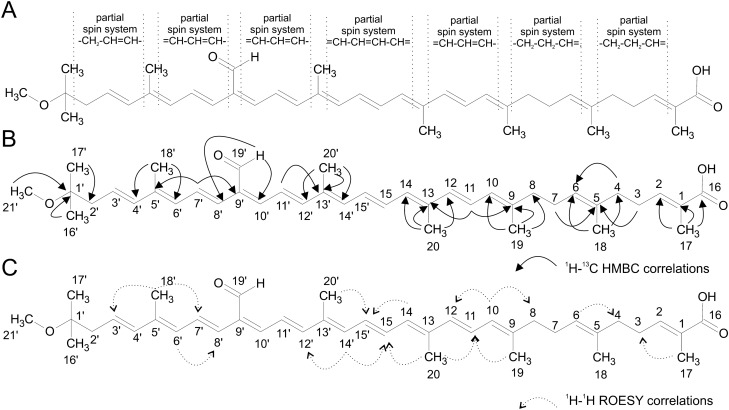
NMR assignment of gemmatoxanthin structure. (**A**) Spin systems based on ^1^H-^1^H COSY spectra. (**B**) Crucial ^1^H-^13^C HMBC and (**C**) crucial ROESY contacts.

The COSY spectrum (Fig. S15-S19) in combination with ^1^H-^13^C HSQC (Fig. S2-S5) allowed us to identify partial spin systems (Fig. 2A), namely one –CH_2_–CH=CH–, three =CH–CH=CH–, one =CH–CH=CH–CH=, and two –CH_2_–CH_2_–CH=. Moreover, the ^1^H NMR also contained an aldehyde signal (δ_C_ 194.2, δ_H_ 9.539), signals of six methyl on a double bond, methoxyl (δ_C_ 48.6, δ_H_ 3.114) and one methyl of a double intensity (δ_C_ 24.7, δ_H_ 1.091). The ^1^H-^13^C HMBC (Fig. S10) further pointed to a carbon atom (C16) with a chemical shift 169.5 ppm indicating the presence of a carboxyl functional group, which was further confirmed by FTIR and MS measurements (see below). The identified structure fragments were partially interconnected by ^1^H-^13^C HMBC depicted in Fig. 2B. As the quaternary carbon C2 and carbon of methine C1 were not observable, we have performed an additional ^1^H-^13^C HMBC experiment using highly ^13^C labeled sample obtained by cultivation in SILEX media. As a result, ^13^C signals were obtained proving the connection of C1 and C2 at 130.9 and 137.0 ppm, respectively (Fig. S20). This connection was further confirmed by the MS/MS data presented below.

The ROESY experiment was used to establish stereochemistry on the double bonds (Fig. S21-S23). The crucial through space interactions detected in the ROESY spectrum are depicted in Fig. 2C. Unfortunately, the arrangement on the double bonds H-8′ to H-12′ was not approved.

### Mass spectrometry fragmentation analysis

**Figure 3:**
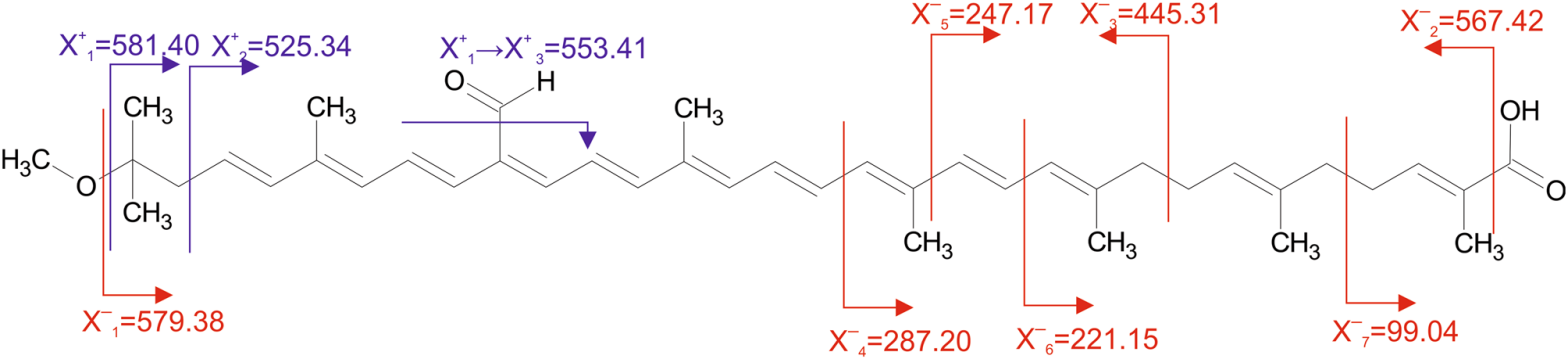
The most prominent MS/MS product ions provided by gemmatoxanthin molecular and demethoxylated ions in the positive (blue) and negative ionization mode (red). For detailed information, refer to the Supplementary Tables S1, S2 and Fig. S24.

The fragmentation experiment performed in the negative ion mode provided a loss of methoxy- group positioned at C1′ (product ion X1^−^, Fig. 3). It was followed by a consecutive CO_2_ loss that formed a product ion at *m*/*z* 535.39. The decarboxylation was also observed directly from the molecular ion (*m*/*z* 567.4213—product ion X2^−^, Fig. 3) that established the presence of a carboxyl group on the NMR unassigned C16 carbon (^13^C-shift 169.5 ppm). Further product ions X_3_^−^ to X_7_^−^ represented the cleavage of the aliphatic chain of the gemmatoxanthin molecule. Although the negative ion MS spectra did not provide a direct neutral loss of the formyl substituent, a consecutive loss of methoxy- (X_1_^+^, Fig. 3) and formyl groups forming a product ion at *m*/*z* 553.41 (X_1_^+^ → X_3_^+^, Fig. 3) was observed in the positive ion MS spectrum. Both the gemmatoxanthin positive and negative ion spectra provided product ions corresponding to the loss of a water molecule (at *m*/*z* 563.3910 and 561.37438, respectively). However, the mechanism of water cleavage remains unexplained. Detailed information on the recorded product ions is provided in the Supplementary information (Tables S1, S2 and Fig. S24).

### Fourier-transform infrared spectroscopy and Raman spectroscopy

**Figure 4:**
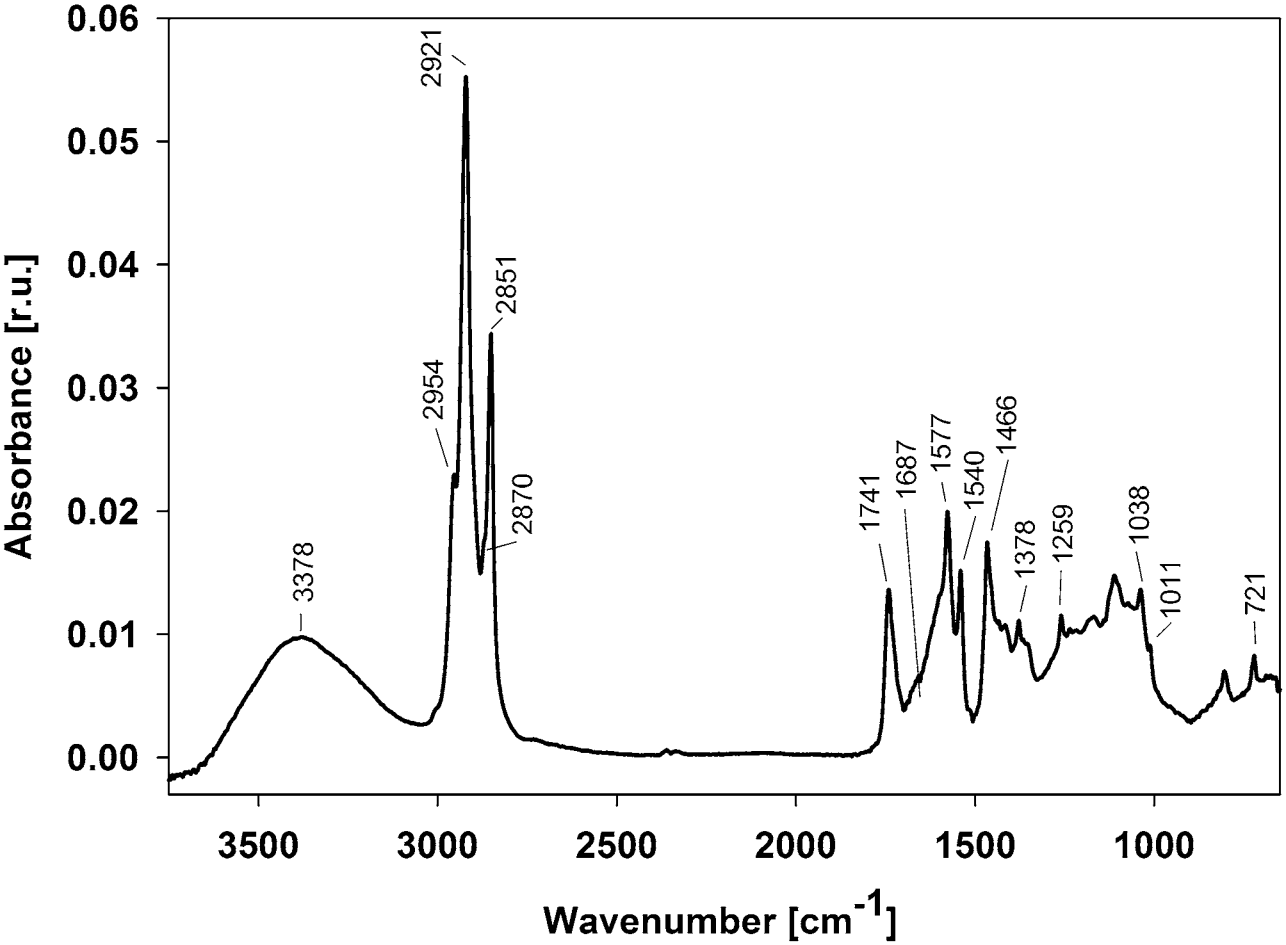
ATR-FTIR spectrum in the (4000–500 cm^−1^, mid-infrared region) of gemmatoxanthin. r.u.- relative units.

The FTIR spectrum of gemmatoxanthin in methanol shows several absorbance peaks ranging from 4000 to 500 cm^−1^ (Fig. 4). Peaks were identified using literature library data^13–16^. A broad absorbance band at a spectrum frequency of 3378 cm^−1^ indicates the presence of a hydroxyl group^16^. An absorbance band at 1741 cm^−1^ corresponds to a C=O stretch of carboxylic acid^17^. A band at 2954 cm^−1^ corresponds to an asymmetric vibration of methyl groups together with a rather weak symmetric vibration at 2870 cm^−1^. Prominent bands at 2921 cm^−1^ and 2851 cm^−1^ correspond to asymmetric and symmetric vibrations of methylene groups^13,16,18^. The vibration band of C–H from the methoxy group (–O–CH_3_) is not visible as it could be overlapped by stronger signals originating from the aforementioned CH_3_ and CH_2_ vibrations^14,16^. The vibration visible as a shoulder at 1687 cm^−1^ indicates the presence of conjugated aldehyde^13^. The symmetric 1577 cm^−1^ and asymmetric 1540 cm^−1^ stretchings of C=C confirms the conjugated system of double bonds in the carbon backbone of the carotenoid^13^. The absence of a vibration band at in region of 3015–3007 cm^−1^ proves that none of the double bonds in the system originate from a *cis* conformation (Fig. 4). The strong band of methylene/ methyl at 1466 cm^−1^ together with weak methyl band at 1378 cm^−1^ (umbrella deformation vibration of C–H of CH_3_) and band at 721 cm^−1^ (methylene rocking vibration) is indicative for long-chain linear aliphatic structures^18^. A weak, but visible, band at 1259 cm^−1^ originates from C–H rocking. A weak band at 1038 cm^−1^ belongs to C–O bending^19^. A weak band at 1011 cm^−1^ is designated as out-of-plane bending and Cα = Cα′ torsion of the backbone chain^20^.

The recorded Raman spectra^21^ contained three characteristic bands typical for carotenoids (Fig. S25). These three bands originate from (1) a rocking vibration of the molecule's methyl component (1015 and 1014 cm^−1^)^22^, (2) from C–C single-bond stretch vibrations (1158 cm^−1^)^23,24^ and (3) C=C stretching vibrations (1525 and 1518 cm^−1^) of the molecule's backbone^22,24,25^. Other Raman bands elicited by the lasers were at 910 and 908 cm^−1^ and considered as C–C symmetric stretch^26^. A vibrational band appearing as a non-resolved band at 1176 and 1175 cm^−1^ belonged to CH_3_ rocking and a weak band at 1591 cm^−1^ (532 nm) and 1585 cm^−1^ (785 nm) was assigned to C=C stretching vibrations^22^.

## Discussion

In the presented study we have identified the structure of the main carotenoid present in the photosynthetic complex of *G. phototrophica* AP64. A combination of NMR and MS experiments has led to the unambiguous structure assignments presented in Figs. 2 and 3, and having a IUPAC-IUBMB semi-systematic name 1′-Methoxy-19′-oxo-3′,4′-didehydro-7,8,1′,2′-tetrahydro-Ψ,Ψ-caroten-16-oic acid.

C40 carotenoids containing a carboxyl group or its variants are more common in the case of algae, fungi and yeast^27^. Only a limited number of photosynthetic prokaryotes are known to have carboxylic carotenoids, for instance, thiothece-484 from *Thiocystis gelatinosa*^28^ or synechoxanthin from cyanobacterium *Synechococcus* sp. PCC 7002^29^. The presence of a carbonyl group or hydroxyl group in the C40 linear carotenoids is commonly known and have been isolated from photosynthetic bacteria. However, the presence of a carboxylic group in the C40 linear carotenoids is rare^27,30,31^.

Gemmatoxanthin’s unique structure is corroborated by a specific MS/MS APCI product ion pattern. The majority of carotenoids provide neutral losses, 92 and 106 Da, corresponding to the electrocyclic elimination of toluene and xylene, respectively^32^. This cleavage was not found in gemmatoxanthin and is very likely due to the presence of a formyl group at C9′ of the conjugated backbone, resulting in a completely different fragmentation pathway. Secondly, although the molecule does not contain any free hydroxyl substituents, it loses a water molecule in both ionization modes and this neutral loss is likely associated with the carboxyl group fragmentation. The consecutive loss of water and CO was reported in the MS/MS spectrum of retinoic acid also lacking hydroxyl and featuring the plausible connection of the carboxyl group to the conjugated linear chain^33^. However, subsequent CO loss in the gemmatoxanthin MS/MS spectrum might be also interpreted as a loss of the formyl functional group. Finally, the CO_2_ loss confirming the presence of the carboxylic group was detected only in the negative ion mode, probably due to the fact the carboxyl loss is manifested as formic acid HCOOH (H_2_O + CO) loss in the positive APCI spectrum as mentioned above.

Using infrared techniques such as FTIR, the key functional groups were identified. A methoxy group exhibits a weak band in the 2860–2800 cm^−1^ region of the infrared spectrum, at a lower frequency than that of the main C–H absorption^14^. The strong absorption of CH_3_ and CH_2_ groups may overlap the absorption region for a methoxy group, which may hinder precise detection^14^. The key functional group vibration at 3378 cm^−1^ corresponds to the hydroxyl group and a band at 1741 cm^−1^ to the C=O stretch. Hence, the carboxyl group characteristic vibration has been proved by FTIR^16^. The absence of the *cis* C = C absorbance band in the region > 3000 cm^−1^ which usually absorbs at ~ 3006 cm^−1^^34^ confirmed an all-*trans* conformation of the carotenoid. Therefore, based on the spectroscopic data analysis, we can conclude the gemmatoxanthin is a linear, all-*trans*, conjugated C_40_ keto carotenoid containing formyl and a carboxylic group.

Resonance Raman spectroscopy showed typical simple spectra that were exclusively dominated by bands at 1525 cm^−1^ assigned to ν(C=C) in phase stretching, 1158 cm^−1^ assigned to ν(C–C) stretching, and 1015 cm^−1^ assigned to a combination of δ(C=CH) methyl in-plane rocking and δ (C−H) out-of-plane bending modes when excited with 785 nm laser and have shown the similar bands at 1518 cm^−1^, 1158 cm^−1^, and 1014 cm^−1^ when excited by 532 nm laser. The wavenumber position of ν(C=C) stretch is usually influenced by the length of the conjugated carbon chain together with changes in the substitution pattern of the chain and by interactions with other constituents in the matrix^35–37^. The linear relationship between the conjugation length *N* and the ν(C=C) Raman band is not always readily followed by β-rings, ketones, conjugated end-cycles containing carotenoids^21,38^. For instance, β-carotene^39,40^ and gemmatoxanthin both possess 11 conjugated double bonds exhibiting the different ν(C=C) Raman band at wavenumber 1517 cm^−1^ and 1525 cm^−1^ respectively. The reason being, β-carotene is a cyclic carotenoid where two conjugated double bonds are in cyclohexane rings resulting lower wavenumber 1517 cm^−1^ while gemmatoxanthin is a linear molecule with a central formyl group C=O connected to the system of conjugated bonds resulting in a shift of higher wavenumber 1525 cm^−1^. No strong bands appearing in the region of 1290–1200 cm^−1^ are indicative for all-trans configuration^41^. The ν(C–C) stretching wavenumber position is similar to carotenoids such as lutein, lycopene, and β-carotene and their mixtures^42^. The δ(C=CH) methyl in-plane rocking and δ (C−H) out-of-plane bending is slightly shifted by ~ 10 cm^−1^ to higher wavenumber ~ 1015 cm^−1^ in gemmatoxanthin than in other known carotenoids with exception of crocetin and cis-Bixin^35^, together with appearance of a band at 1175–76 cm^−1^ may be indicative for contamination with some other carotenoid.

Carotenoids, owing to their electron-rich polyene chain, are known to photodegrade or isomerize upon exposure to light, heat, oxygen, acids or alkaline base^43,44^. Therefore, to avoid the photooxidation, carotenoids were measured in dark, are kept in low temperature and in the inert environment (under nitrogen). However, during the NMR measurement, the sample was measured in DMSO-*d*_*6*_ at 30 °C without excess of light. Under these conditions the sample was stable for three days and between NMR measurements, the sample was stored at − 80 °C. We observed substantial degradation of the sample after the third round of freezing/melting.

The presence of the carbonyl-containing carotenoids is randomly distributed in different families of anoxygenic phototrophic bacteria where keto- carotenoids are much more common than carotenoids containing an aldehyde group. Spheroidenone, one of the most studied ketocarotenoids is the main light harvesting carotenoid in phototrophic species belonging to the ‘so called’ Roseobacter clade, which represents a very common group of marine bacteria^45^. It was speculated that shift from spheroidene to spheroidenone, which occurs in *Rhodobacter sphaeroides* under aerobic conditions, may help to quench potentially dangerous singlet oxygen^46^. Similar characteristics may have also predicted in the carotenoid bacteriorubixanthinal present in the photosynthetic complexes of *Erythrobacter* species^47^. Numerous studies have been published on the energy transfer efficiency of carbonyl carotenoids to the BChl *a* in photosynthetic complexes^47,48^. The energy transfer efficiency of rhodopinal to BChl *a* found to be ~ 100%^49^ and similarly, for okenone the energy transfer efficiency is ~ 95%^50^.

**Figure 5:**
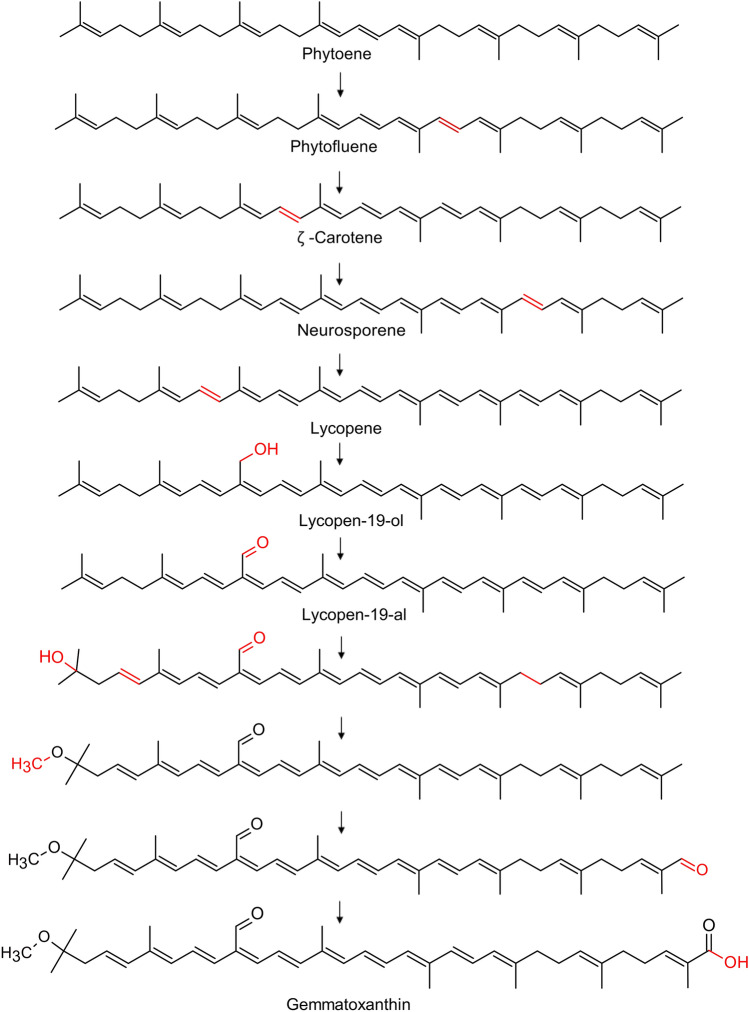
Tentative biosynthetic pathway of gemmatoxanthin. The biosynthesis of the gemmatoxanthin pathway begins from phytoene and lycopene^9^. Lycopene undergoes modification by the hydroxylase/oxidase activity of enzymes (genes are not yet known) to form lycopen-19-al which was tentatively predicted in *Lamprocystis roseopersicina* belonging to the *Chromatiaceae* family^50,51^. The *cruF* gene and *crtF* gene are probably responsible for the addition of terminal methoxy group in the pathway^8^. We hypothesize that the 1′ carbon of methyl group undergoes modification by the hydroxylase/oxidase enzyme encoded by *cruH* gene yielding carboxylic acid, which is already known in synechoxanthin pathway^29,54^. Two unidentified genes similar to *cruH* gene was found in the genome of the *G. phototrophica* AP64 using tblastn showing 36.607% similarity (GEMMAAP_15370) and 23.034% identity (GEMMAAP_15125).

The biosynthetic pathway of the novel carotenoid is another important issue. As demonstrated previously, *G. aurantiaca* contains a biosynthetic pathway of (2*S*,2′*S*)-oscillol 2,2′-di-(α-L-rhamnoside) with lycopene as its intermediate^9^. It is, therefore, plausible to assume that gemmatoxanthin synthesis begins from phytoene and lycopene (Fig. 5). Phytoene, the first C_40_-carotene, is converted to lycopene by incorporating double bonds. This 4-step reaction is carried out by phytoene dehydrogenase (phytoene desaturase) encoded by *crtI* (GEMMAAP_12150) responsible for extending the conjugated double bonding. Lycopene undergoes hydroxylation and then oxidation at the C-19 position forming thus lycopen-19-al probably by the hydroxylase/oxidase activity of enzymes. This hypothesis is based on the fact that lycopene-19-al was first isolated from *Lamprocystis roseopersicina* belonging to the *Chromatiaceae* family and this pathway was previously tentatively predicted^50,51^. A similar pathway has also been predicted for lycopene-20-al, but as yet, no enzyme has been identified for carrying out the hydroxylation/oxidation reaction either at C-19 or the C-20 carbon position^52^. Furthermore, the addition of the terminal methoxy group is probably initiated by a carotenoid 1,2-hydratase encoded by *cruF* gene. Subsequent to this, the methyl is transferred by a carotenoid O-methyltransferase, which is likely encoded by *crtF* gene present in the PGC^8^. We can hypothesize that the methyl group of 1′ carbon undergoes a hydroxylation and oxidation reaction yielding carboxylic acid, which is already known for synechoxanthin^29^ and torulahadrin carotenoids^53^ isolated from the unicellular Cyanobacterium *Synechococcus* sp. strain PCC 7002 and from the yeast *Rhodotorula*, respectively. Synechoxanthin is an aromatic carotenoid, the pathway of which is derived from β-carotene. One of the intermediates of the synechoxanthin pathway consists of β,χ-carotene ring at one end and renierapurpurin (χ,χ-carotene) at the other end of the carotenoid backbone. The methyl group of renierapurpurin is eventually oxidized to carboxylic acid by a hydroxylase/oxidase encoded by *cruH* gene^29,54^. In order to confirm that AP64 has similar enzymes carrying out a similar hydroxylation/oxidation reaction, we searched the CruH protein sequence against the genome of *G. phototrophica* AP64 (CP011454.1) using tblastn. We found two unidentified proteins showing 36.61% similarity (GEMMAAP_15370) and 23.03% identity (GEMMAAP_15125) that may be responsible for methyl conversion to carboxyl in the gemmatoxanthin pathway. Similarly, torularhodin is a C40 carotenoid having a linear carboxylic acid at one end and the other end consists of a 6-membered ring. Torulene, an initial precursor of the pathway, then undergoes hydroxylation and oxidation to yield carboxylic acid in a similar manner as described for synechoxanthin. The enzymes involved in the torularhodin pathway are, unfortunately, not known^53,55^.

In summary, gemmatoxanthin represents a novel natural compound. To our knowledge, this is the first linear C40 carotenoid that contains a carboxyl group at one end of the molecule, a methoxy substituent at the other end and a central formyl group connected to the system of conjugated double bonds, all of which strongly affects the spectral characteristics of the molecule.

## Methods

### Cultivation of G. phototrophica AP64

The strain *G. phototrophica* AP64^5^ was grown on solid agar media containing per litre 0.5 g yeast extract, 0.5 g peptone, 0.2 g pyruvate, 0.2 g glucose, 1 g K_2_HPO_4,_ pH 7.7, 15 g of Bacto™ Agar, and 1 ml of modified SL8 trace metal solution (190 µg CoCl_2_.6H_2_O, 5.2 mg Na_2_-EDTA, 24 µg NiCl_2_.6H_2_O, 17 µg CuCl_2_.2H_2_O, 70 µg ZnCl_2_, 1.8 mg SrCl_2_.6H_2_O, 20.3 mg MgCl_2_, 62 µg H_3_BO_3_ per ml) and 1 ml vitamins (200 µg B1, 20 µg B3, 10 µg B7, 10 µg B12 per ml). Cells were streaked on agar media plates and incubated for approx. 2 weeks in microaerophilic conditions (10% O_2_ + 90% N_2_) at 28 ± 1 °C, and pH-7.7 in the dark. The colonies were scraped from the agar media plates, using a plastic scraper, into a suitable tube, pelleted, washed and stored at − 20 °C until needed.

### Extraction and purification of photosynthetic membranes

The harvested cells (9–10 g wet) were re-suspended in 20 mM, Tris. Cl, 50 mM NaCl pH 8.0 and homogenized thoroughly with a few grains of DNAse and a few mg of MgCl_2_. The cells were broken by passage three times through an Emulsiflex-CS cell disrupter and unbroken cells removed by low-speed centrifugation (10 min, 12,000 g, 4 °C). The membranes were pelleted by ultra-centrifuge Beckman L8-55 M equipped with the fixed angel rotor Ti-55.2 (180,000×g, 2 h at 4 °C) and gently resuspended in 20 mM, Tris. Cl pH 8.0 and adjusted to an optical density (OD) =  ~10 cm^−1^ at the Qx band (~ 580). The re-suspended membranes were then solubilized with 2% n-dodecyl β-D-maltoside (DDM), 0.2% Triton X-100 for 60 min, stirred at room temperature in the dark and then centrifuged to remove any un-solubilized material. The photosynthetic complex was then purified by layering on top of a stepwise sucrose gradient and run overnight (208,000 × g, 16 h at 4 °C). The gradient comprised sucrose dissolved in TD buffer (20 mM Tris.Cl pH 8.0, 0.02% DDM) layered in 0.2 M steps (1.6 to 0.4 M). The following day the band containing photosynthetic complex was carefully removed from the gradient and loaded on to a gravity Q-Sepharose ion-exchange column. The column was pre-equilibrated with 20 mM, Tris. Cl pH 8.0 and after loading the sample was washed with copious amounts of TD buffer to remove sucrose and any non-specifically bound proteins. The photosynthetic complex was eluted by progressively increasing the NaCl concentration in the TD buffer. The resulting fractions were pooled and concentrated prior to gel filtration. Gel filtration was performed using an XK16/Superdex S300 column (GE Healthcare) at a flow rate of 0.5 ml min^−1^ at 22 °C. The collected fractions with the best optical ratio were pooled and concentrated as required. The final photosynthetic complex pool typically had an A817/A260 =  ~1.4 to 1.3.

### Gemmatoxanthin purification and HPLC–PDA analysis

Pigments were extracted twice in 100% methanol (HPLC grade, VWR Czech Republic) from wet cells or purified complexes. The extracted pigments were centrifuged at top speed for 3 min in the Eppendorf centrifuge and the supernatant evaporated to dryness under a stream of nitrogen. The dried sample was dissolved in 50 μl of HPLC grade methanol mixed with 5 µl of 25% of ammonium acetate buffer and 20 μl was injected into a SCL-40 HPLC (Nexera series, Shimadzu, Japan) equipped with SPD-M40 PDA detector. The pigments were separated using a reverse-phase analytical C8 column (Kinetex, 2.6 µm, 100 Å, 150 × 4.6 mm column, Phenomenex, USA) heated at 40 °C and the following mobile phase was employed: 25% 28 mM ammonium acetate in water with 75% methanol (solvent A) and 100% methanol (solvent B) at a flow rate of 0.8 ml/min. Gradient is as follows: A/B 0/100 (23 min), 0/100 (25 min),100/0 (27 min), and 100/0 (28 min). Eluting pigments peaks were monitored at spectral range of 250–800 nm and was collected manually. Samples were handled in ice under the dim light condition to minimize its oxidation and isomerization.

### Spectroscopic analysis

Dry and purified carotenoid was dissolved in hexane or methanol, and its absorbance spectrum was measured and recorded in UV 2600 spectrophotometer (Shimadzu, Japan). The reduction was carried out by adding a few crystals of NaBH_4_ in purified carotenoid dissolved in 1 ml of methanol^56^. Absorbance spectra were measured from 250 to 700 nm, with a resolution of 0.5 nm in 1 cm quartz cuvettes against the pure solvent as a blank.

### HPLC-APCI-HRMS of carotenoids

Carotenoid samples, extracted and purified from 1 gm of wet cells, were analysed on a Dionex UltiMate 3000 UHPLC + (Thermo Scientific, Sunnyvale, CA, USA) equipped with a diode-array detector. Purity and determination of *m*/*z* of carotenoids was performed on a reversed phase C18 column (Kinetex, 150 × 4.6 mm, 2.6 µm, Phenomenex, Torrance, CA, USA) using H_2_O (A)/methanol (B) both containing 0.1% HCOOH as a mobile phase with the flow rate of 0.6 ml min^−1^. The gradient was as follows: A/B 85/15 (0 min), 85/15 (in 1 min), 0/100 (in 20 min), 0/100 (in 25 min) and 85/15 (in 30 min). The chromatographic instrument and conditions for measuring positive and negative mass spectra were identical. To acquire the fragmentation spectra in positive mode the HPLC was connected to an Impact HD high-resolution mass spectrometer (Bruker, Billerica, Massachusetts, USA) equipped with atmospheric pressure chemical ionization (APCI) ionization source. The following APCI probe settings were used: drying temperature, 250 °C; drying gas flow, 12 l min^−1^; nebulizer gas pressure, 3 bar; capillary voltage, 4.0 kV; endplate offset, 500 V. The APCI MS spectra were collected in the range *m*/*z* 20–2000 with the spectral rate of 3 Hz. The MS/MS experiments were performed using molecular ion at *m*/*z* 613.4106 Da [M + H]^+^ and demethoxylated ion at *m*/*z* 581.3844 Da as precursor ions with colision energies 35 and 28 eV, respectively. The calibration of the instrument was performed using sodium formate clusters at the beginning of each analysis. The molecular formulas of obtained molecular peaks and fragments were calculated using Smart Formula function in Bruker Compass Data Analysis software (version 5.1). Negative ion mass spectra were acquired by a Q Exactive Plus Orbitrap HRMS (Thermo Fisher Scientific, San Jose, CA, USA) equipped with an APCI ion source operated at -5 kV spray voltage; capillary temperature, 250 °C, sheath gas at 30 au, aux gas at 10 au, spare gas at 0 au; probe temperature, 400 °C and S-Lens level at 60 au. Full scan HRMS spectra were recorded in a mass range of *m*/*z* 50–750 at 70,000 resolution (R, *m*/*z* 200); scan rate, ± 3 Hz; automatic gain control (AGC) target, 3 × 10^6^ and maximum ion injection time (IT), 100 ms. The negative ion MS/MS experiments using molecular ion at *m*/*z* 611.4106 Da [M-H]^−^ and demethoxylated ion at *m*/*z* 579.3844 Da as precursor ions were conducted at R = 17,500 (*m*/*z* 200), automatic gain control target, 2 × 10^5^ and maximum ion injection time (IT), 100 ms; isolation window, 3 m/*z*; stepped normalized collision energy, 20, 35, 45. For the accurate mass measurements, the lock mass at *m*/*z* 301.9981 (2,4,6-Tris(trifluoromethyl)-1,3,5 triazene, 25 µmol L^−1^, 2 µl min^−1^) was used. The data were processed using a 4.0 Xcalibur™ software (Thermo Fisher Scientific, San Jose, CA, USA).

### Fourier transform infrared spectroscopy (FTIR) and Raman spectroscopy

10 µg of pure carotenoid was dissolved in 10 µl of methanol and 1 µl was deposited onto ATR crystal via Hamilton syringe. After evaporation of the solvent, the Fourier transform spectra were obtained with a Nicolet IS10 (Thermo Nicolet) spectrometer equipped with Smart iTR accessory with the installed ZnSe ATR crystal. Absorbance spectra were collected in the spectral range from 600 to 4000 cm^−1^, at a spectral resolution of 4 cm^−1^; 32 scans were co-added. A Norton-Beer apodization function was used, with a zero-filling factor of 2. The background was measured using pure methanol after evaporation. Omnic software (Nicolet) was used for measurement and data processing.

DXR2 Raman Microscope with 532 and 785 nm excitation (Thermo Fisher Scientific, USA) was employed where the confocal measurement was through the glass while direct measurement was performed on a thin film on a metal slide. 10 µg of pure carotenoid was dissolved in methanol. We used 10 mW on the sample for 532 nm excitation laser, with laser spot approx. 1 μm. Spectra were collected with one second exposure time and 300 exposures for one Raman spectrum. For 785 nm excitation laser, we used 30 mW on the sample with laser spot approx. 1.5 μm. Spectra were collected with one second exposure time and 30 exposures for one Raman spectrum. Spectra were recorded over a wavenumber range of 50–3500 cm^−1^. Omnic software (Nicolet) was used for measurement and data processing.

### Gemmatoxanthin purification for NMR analysis

For NMR studies, the *G. phototrophica* AP64 cells were grown on modified agar media containing per liter: 0.5 g of ^13^C labelled glucose, 0.5 g yeast extract, 1 g K_2_HPO_4_, 15 g of Bacto™ Agar, and 1 ml of modified SL8 trace element solution as mentioned previously. The incorporation of ^13^C glucose displayed low labelling in the gemmatoxanthin. Therefore, in order to obtain highly ^13^C labeled substituted gemmatoxanthin, the second batch of *G. phototrophica* AP64 cells was grown with ^13^C labelled SILEX *E. coli* media (0.5 g L^−1^) (Silantes GmbH, Munich, Germany) instead of ^13^C labelled glucose. Approx. 15 g (wet weight) of cells scraped from agar plates were used as the starting material for the extraction and purification of the complexes as mentioned earlier. Pigments were extracted in 100% methanol from the purified complexes until it becomes colorless and was centrifuged. The supernatant was concentrated under a nitrogen stream. Carotenoids were purified using a Shimadzu Prominence-*i* high-performance liquid chromatography system equipped with PDA detector. The purification of carotenoid was carried out into two steps: reverse phase semi-preparatory C8 column (250 × 10 mm, Luna 5 μm, Phenomenex Inc., USA) heated at 40 °C was eluted with the following mobile phases: 25% HPLC water with 75% methanol (solvent A) and 100% methanol (solvent B) at a flow rate of 3.0 ml min^−1^ using a gradient as follows: A/B 75/25 (0.01 min), 50/50 (in 16 min), 50/50 (in 23 min), 0/100 (in 40 min), 0/100 (in 42 min), 75/25 (in 43 min), 75/25 (in 56 min). Eluting pigments were monitored at 490 nm wavelength using PDA. The gemmatoxanthin peak was collected and evaporated completely under the stream of nitrogen. In the second and final step of purification, partially purified and dried carotenoid sample was dissolved in methanol and was injected on HPLC semi-preparative phenyl column (8 × 250 mm, Reprosil 100 phenyl 5 μm, Watrex) eluted with HPLC grade water (A) and 100% methanol (B) at a flow rate of 2.0 ml min^−1^ using following gradient: A/B 20/80 (0.01 min), 10/90 (10 min), 5/95 (20 min), 0/100 (21 min), 0/100 (26 min) and 20/80 (28 min).

### Nuclear magnetic resonance (NMR) analysis

Bruker Avance III 700 MHz spectrometer equipped with TCI CryoProbe (700.13 MHz for ^1^H, 176.05 MHz for ^13^C, Bruker Biospin GmbH, Rheinstetten, Germany) was used to acquire NMR spectra in DMSO-*d*_6_ at 303.1 K. The solvent signals were used as an internal standard (*δ*_H_ 2.499 ppm and *δ*_C_ 39.5 ppm). The ^1^H NMR, COSY, ^1^H-^13^C HSQC, ^1^H-^13^C HMBC, *J*-resolved, and ROESY spectra were measured using the standard manufacturer’s software. Pre-saturation sequence was used to eliminate strong signals of residual signal in DMSO-*d*_*6*_ and water signal in ^1^H NMR, COSY, and ^1^H-^13^C HSQC due to a low concentration of the sample. The ^1^H NMR spectrum was zero-filled to fourfold data points, and line broadening was applied (0.3 Hz) before the Fourier transformation. Protons were assigned by COSY, and the assignment was transferred to carbons by HSQC. The chemical shifts are given in the scale (ppm) and coupling constants are given in Hz. The digital resolution (0.3 Hz for ^1^H) allowed us to present the proton chemical shifts to three and coupling constant to one decimal places, respectively. The carbon chemical shifts are readouts from ^1^H-^13^C HSQC and ^1^H-^13^C HMBC and are reported to one decimal place.

## Supplementary Information

Supplementary Information.
