# Supplementary material for: Structure elucidation of the novel carotenoid gemmatoxanthin from the photosynthetic complex of Gemmatimonas phototrophica AP64
Source: Sci Rep. 2021 Aug 5;11:15964. doi: 10.1038/s41598-021-95254-6 (PMC8342508; doi:10.1038/s41598-021-95254-6)
Supplement: Supplementary file 1 — Supplementary Information. [file 41598_2021_95254_MOESM1_ESM.pdf]

Electronic Supplementary Material

Article Title: Structure elucidation of the novel carotenoid gemmatoxanthin from the photosynthetic complexes of *Gemmatimonas phototrophica* AP64

Authors: Nupur[1], Marek Kuzma[2], Jan Hájek[1,3], Pavel Hrouzek[1,3], Alastair T. Gardiner[1], Martin Lukeš[1], Martin Moos[4], Petr Šimek[4], Michal Koblížek[1*]

Affiliation: [1]Centre Algatech, Institute of Microbiology, The Czech Academy of Sciences (CAS), 379 81, Třebon, Czechia. [2]Laboratory of Molecular Structure Characterization, Institute of Microbiology of the Czech Acad Sci, Prague, Czechia. [3]University of South Bohemia, Faculty of Science, Branišovská 1760, Ceské Budějovice, Czechia. [4] Biology Centre of the Czech Academy of Sciences, Branišovská 1160/31, České Budějovice 370 05, Czechia.

Corresponding Author: koblizek@alga.cz

m/z	Summ formula	error		annotation
581.4023	$C_{40}H_{52}O_3$+H^+	-5.9ppm	methoxy- loss	X^+_1
563.3910	$C_{40}H_{50}O_2$+H^+	-4.7ppm	water loss	not explained
553.4113	$C_{39}H_{52}O_2$+H^+	-13.2ppm	methoxy- & formyl-loss	X^+_1 =>X^+_3
525.3387	$C_{36}H_{44}O_3$+H^+	-4.5ppm	terminal $C_5H_{10}O$ loss	X^+_2
399.2691	$C_{29}H_{34}O$+H^+	-2.2ppm	not assigned	not assigned
359.2369	$C_{26}H_{30}O$+H^+	+0.2ppm	not assigned	not assigned
341.2288	$C_{26}H_{28}$+H^+	-7.2ppm	not assigned	not assigned

Table S1: The major HRMS-APCI products ion in positive mode

m/z	Summ formula	error		annotation
579.38459	$C_{40}H_{50}O_3^-$	0.4 ppm	methoxy- loss	X^-_1
561.37438	$C_{40}H_{48}O_2^-$	1.0 ppm	water loss	not explained
535.39335	$C_{39}H_{50}O^-$	-2.2 ppm	CO_2 loss	X^-_1=>X^-_2
445.3105	$C_{31}H_{40}O_2^-$	-1.7 ppm		X^-_3
287.2021	$C_{19}H_{26}O_2^-$	1.5 ppm		X^-_4
247.1700	$C_{16}H_{22}O_2^-$	-1.4 ppm		X^-_5
221.1549	$C_{14}H_{20}O_2^-$	-0.8 ppm		X^-_6
197.0970	$C_{14}H_{12}O^-$	-0.8 ppm	not assigned	not assigned
187.1126	$C_{13}H_{14}O^-$	-1.2 ppm	not assigned	not assigned
171.0815	$C_{12}H_{10}O^-$	-0.2 ppm	not assigned	not assigned
99.0450	$C_5H_6O_2^-$	-3.4 ppm		X^-_7

Table S2: The major HRMS-APCI products ions in negative mode

Supplementary Figures

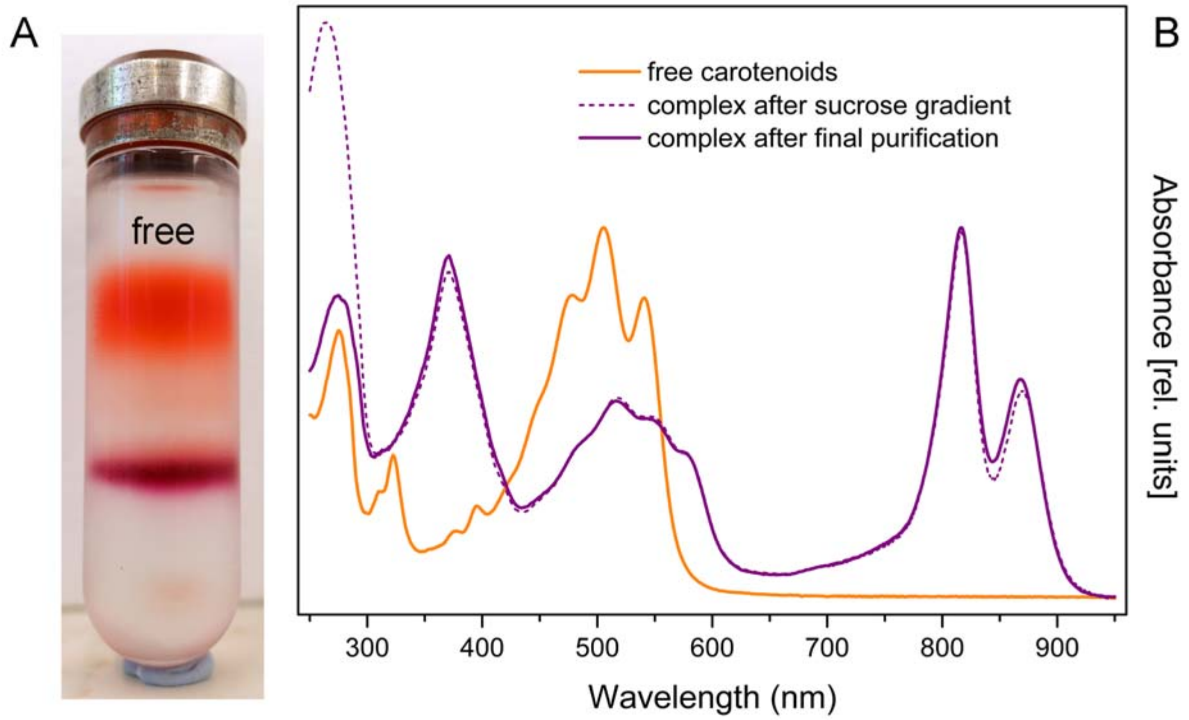

Figure S1: (A) A picture of the sucrose gradient used to separate the photosynthetic complex (purple band) from the free carotenoid fraction (orange band). (B) The resulting UV-VIS absorption spectra (in TD buffer) of the carefully extracted free carotenoid fraction (orange) and the complex from the gradient (purple dashes) as well the spectra of the final complex sample (purple) after purification to homogeneity and gemmatoxanthin extraction.

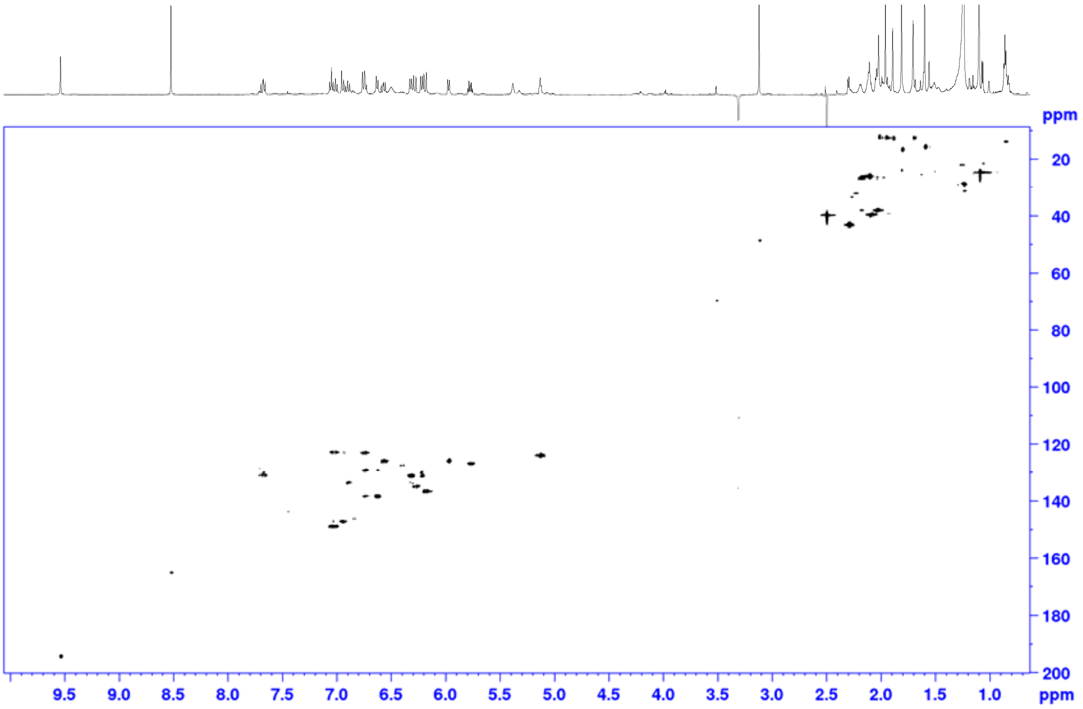

Figure S2: A ^{1}H-^{13}C HSQC spectrum of gemmatoxanthin (700.13 MHz for ^{1}H, 176.05 MHz for ^{13}C, DMSO-d_6, 303.1 K)

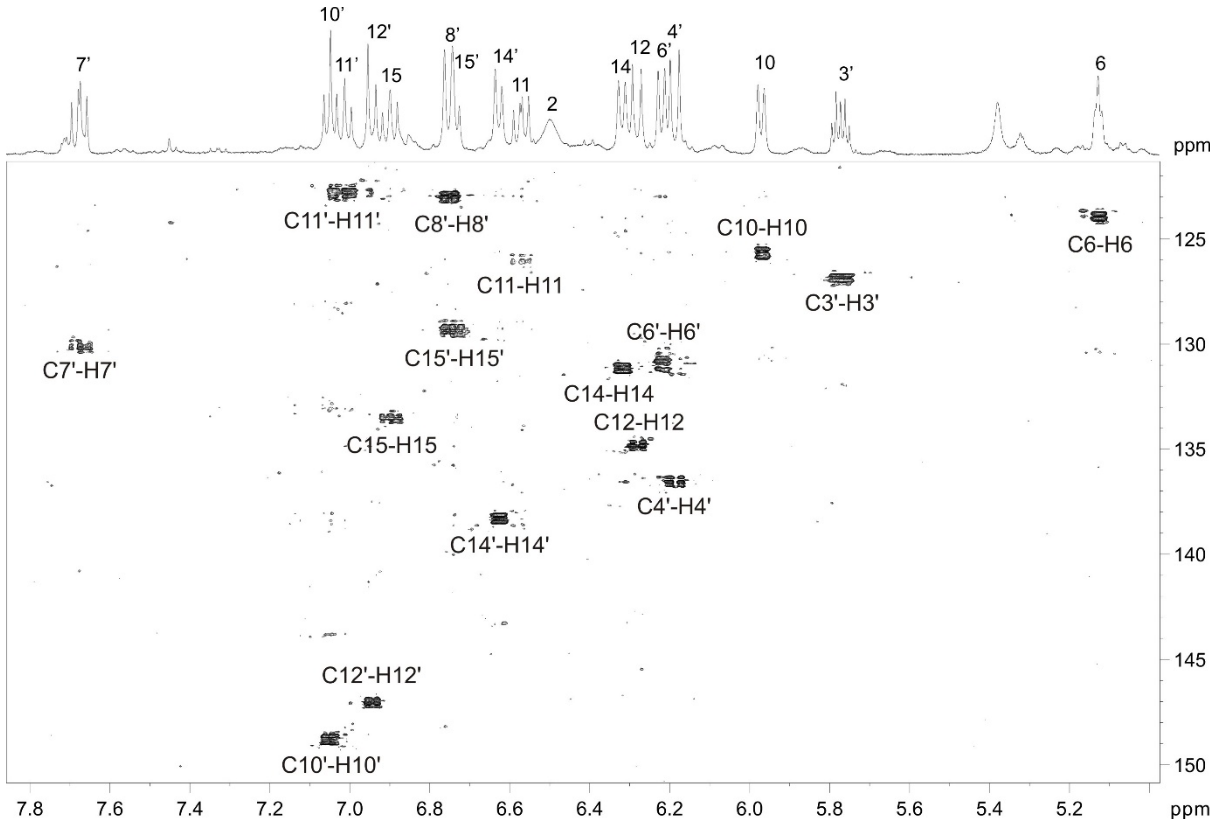

Figure S3 : A detail of the HSQC spectrum of gemmatoxanthin (700.13 MHz for ^{1}H, 176.05 MHz for ^{13}C, DMSO-d_6, 303.1 K).

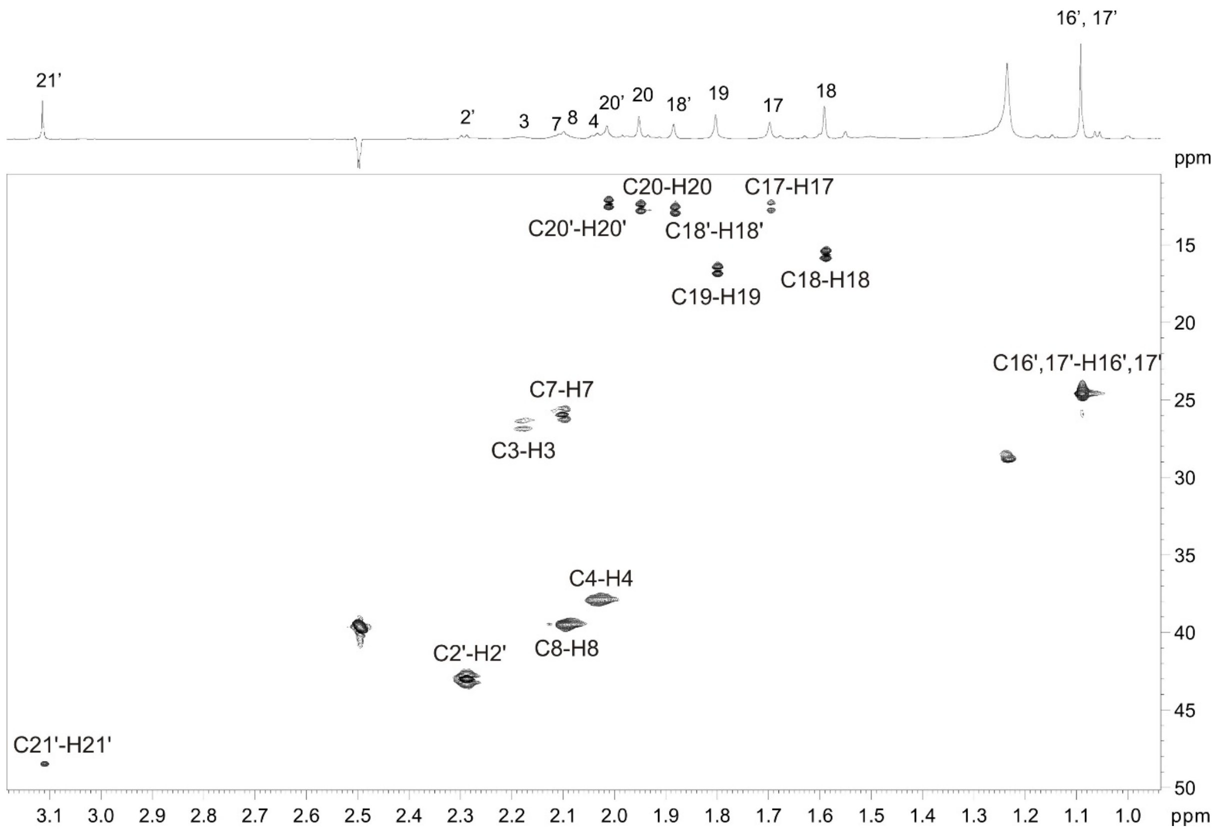

Figure S4: A detail of the HSQC spectrum of gemmatoxanthin (700.13 MHz for ^{1}H, 176.05 MHz for ^{13}C, DMSO-d_6, 303.1 K).

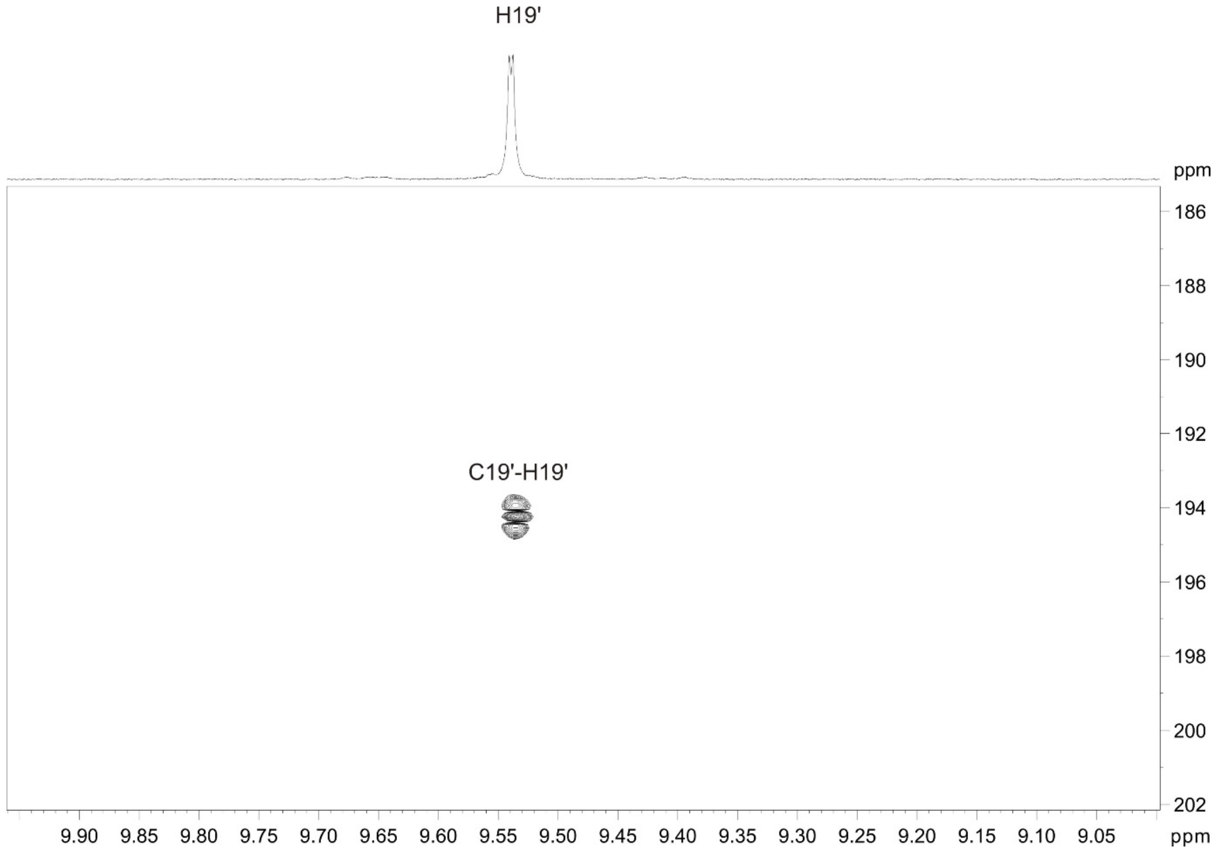

Figure S5: A detail of the HSQC spectrum of gemmatoxanthin (700.13 MHz for ^{1}H, 176.05 MHz for ^{13}C, DMSO-d_6, 303.1 K).

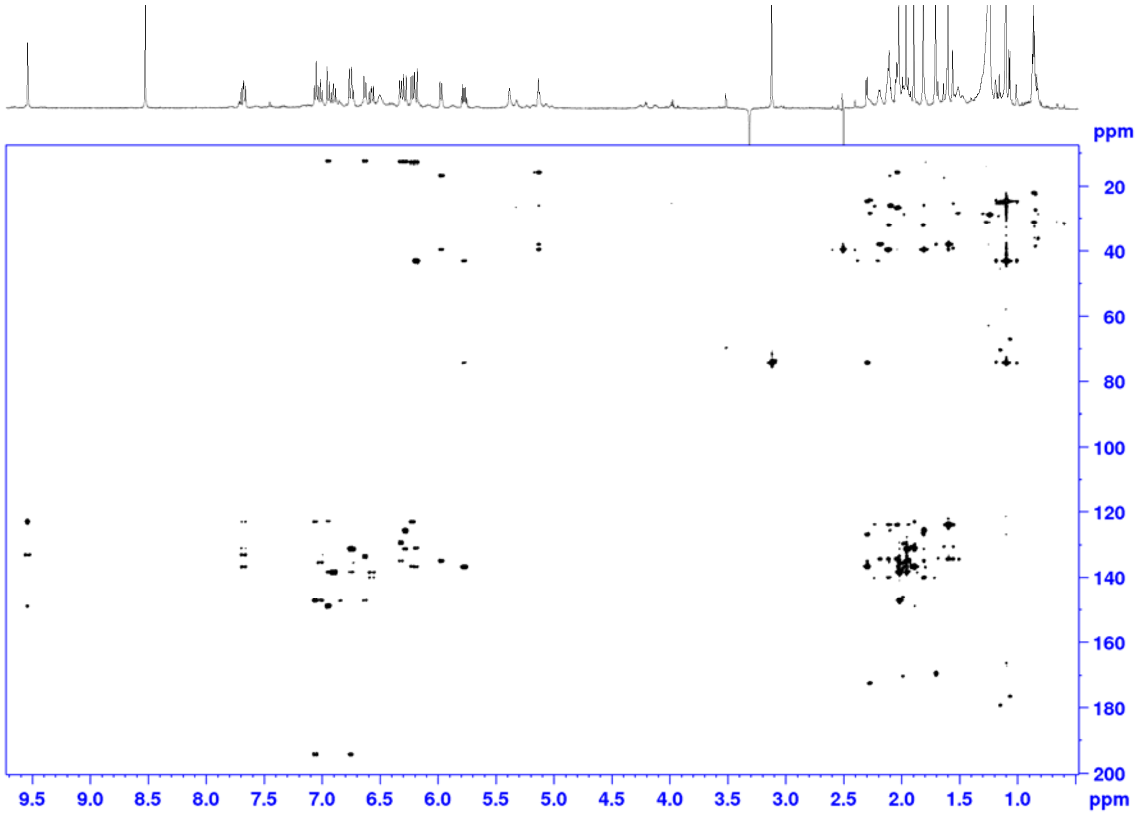

Figure S6: A ^{1}H-^{13}C HMBC spectrum of gemmatoxanthin (700.13 MHz for ^{1}H, 176.05 MHz for ^{13}C, DMSO-d_6, 303.1 K)

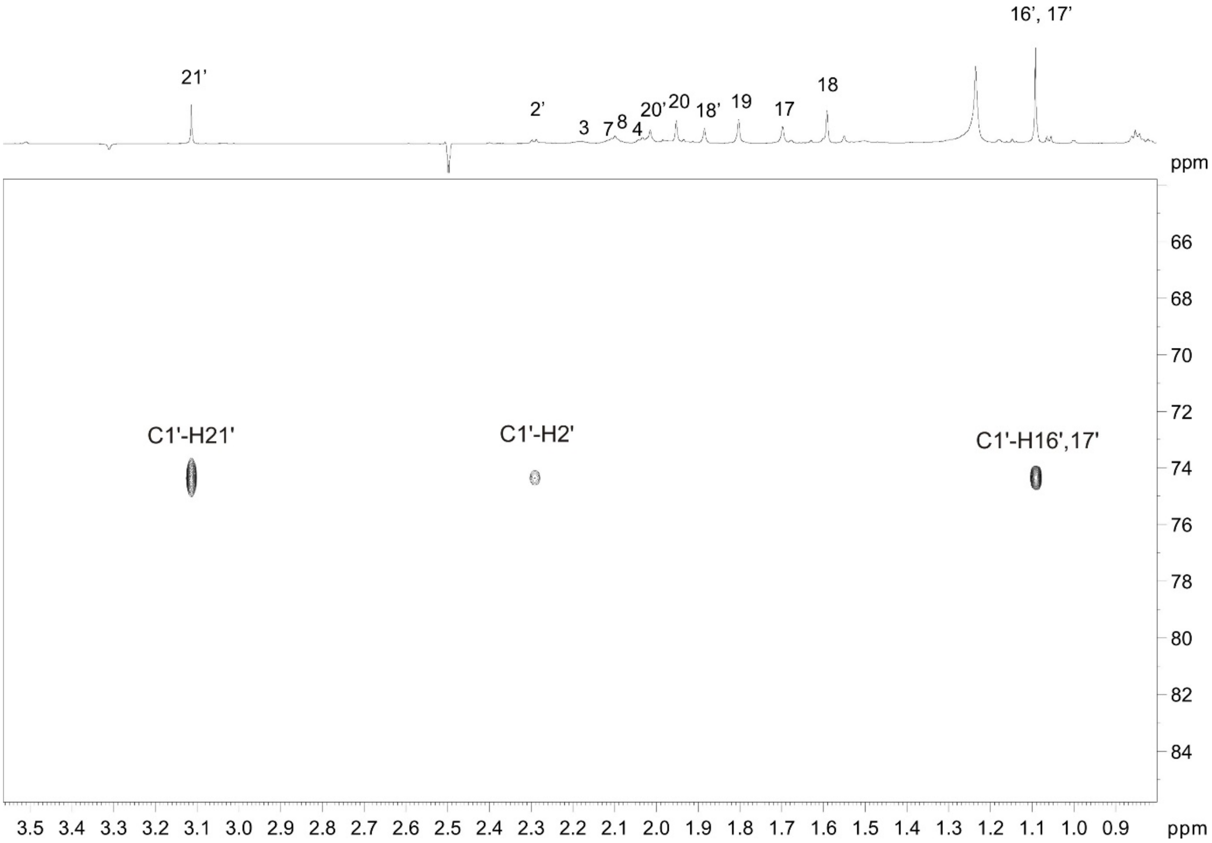

Figure S7: A detail of the HMBC spectrum of gemmatoxanthin (700.13 MHz for ^{1}H, 176.05 MHz for ^{13}C, DMSO-d_6, 303.1 K).

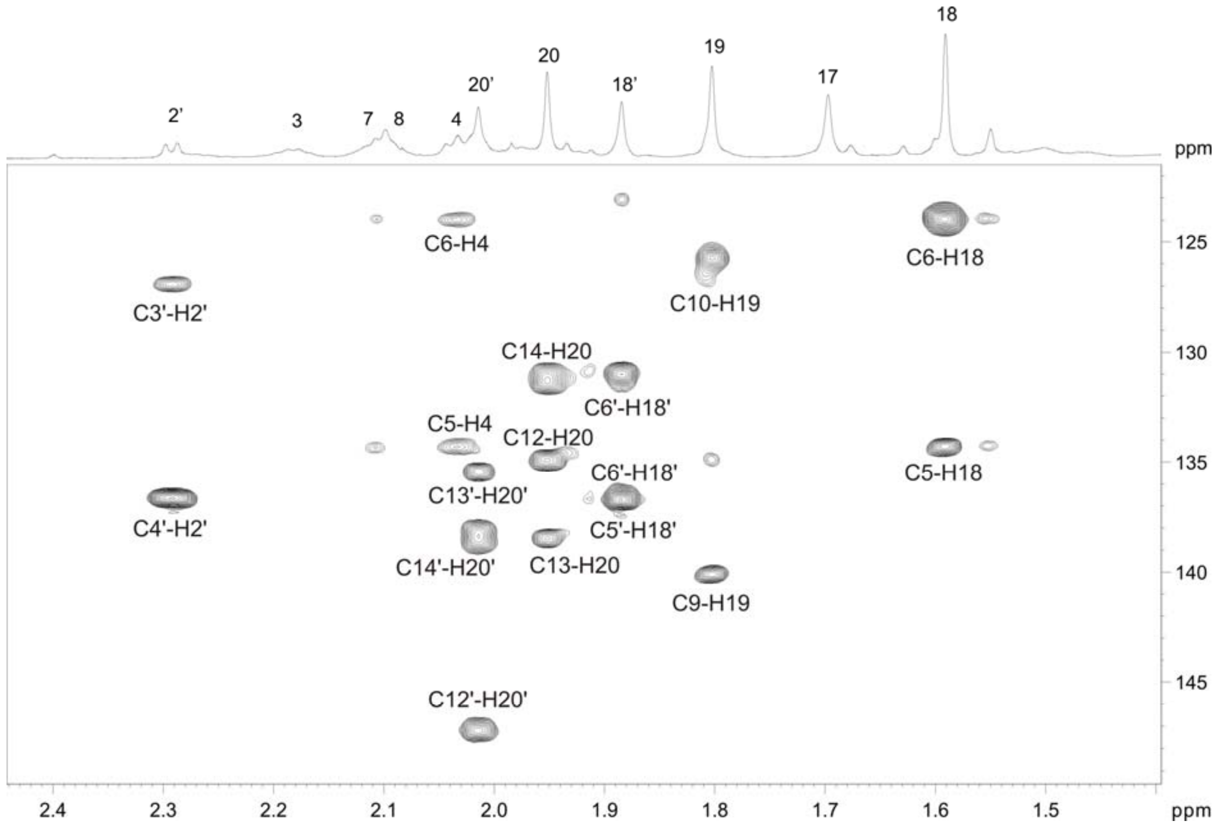

Figure S8: A detail of the HMBC spectrum of gemmatoxanthin (700.13 MHz for ^{1}H, 176.05 MHz for ^{13}C, DMSO-d_6, 303.1 K).

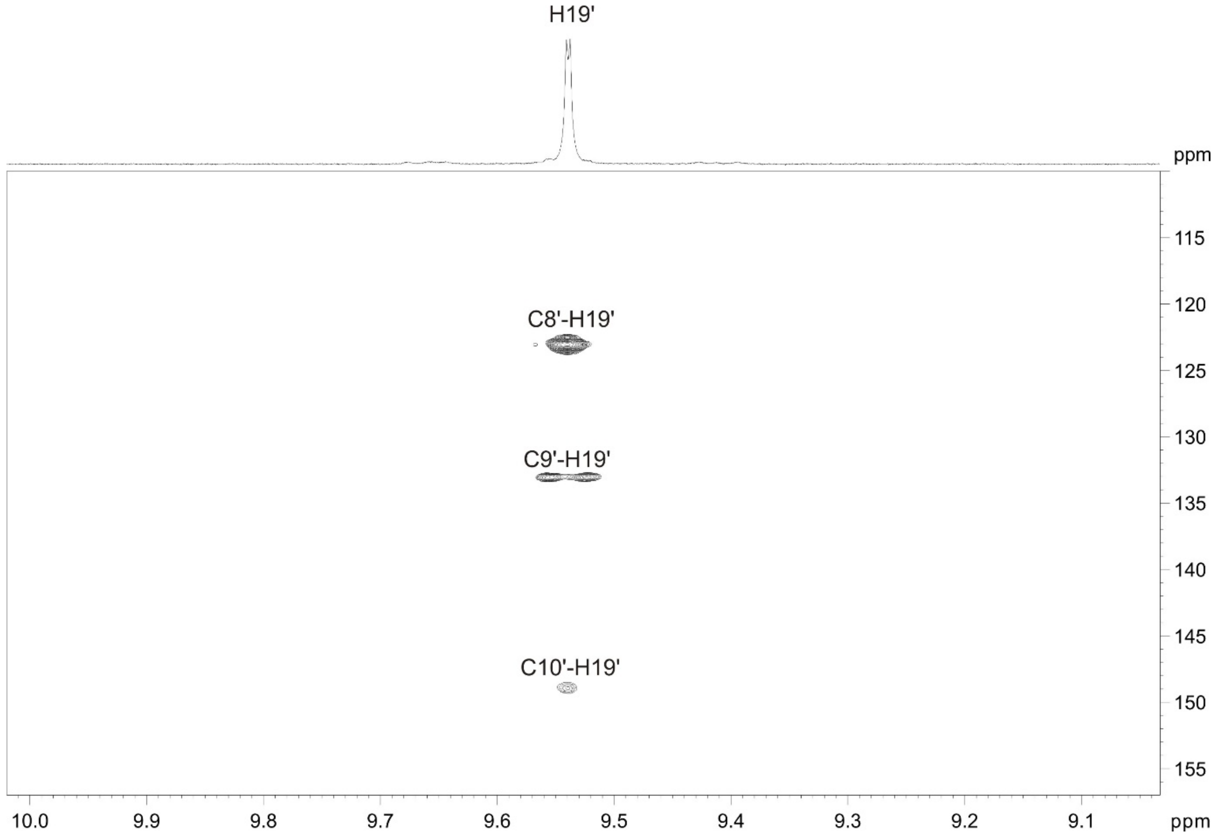

Figure S9: A detail of the HMBC spectrum of gemmatoxanthin (700.13 MHz for ^{1}H, 176.05 MHz for ^{13}C, DMSO-*d*$_6$, 303.1 K).

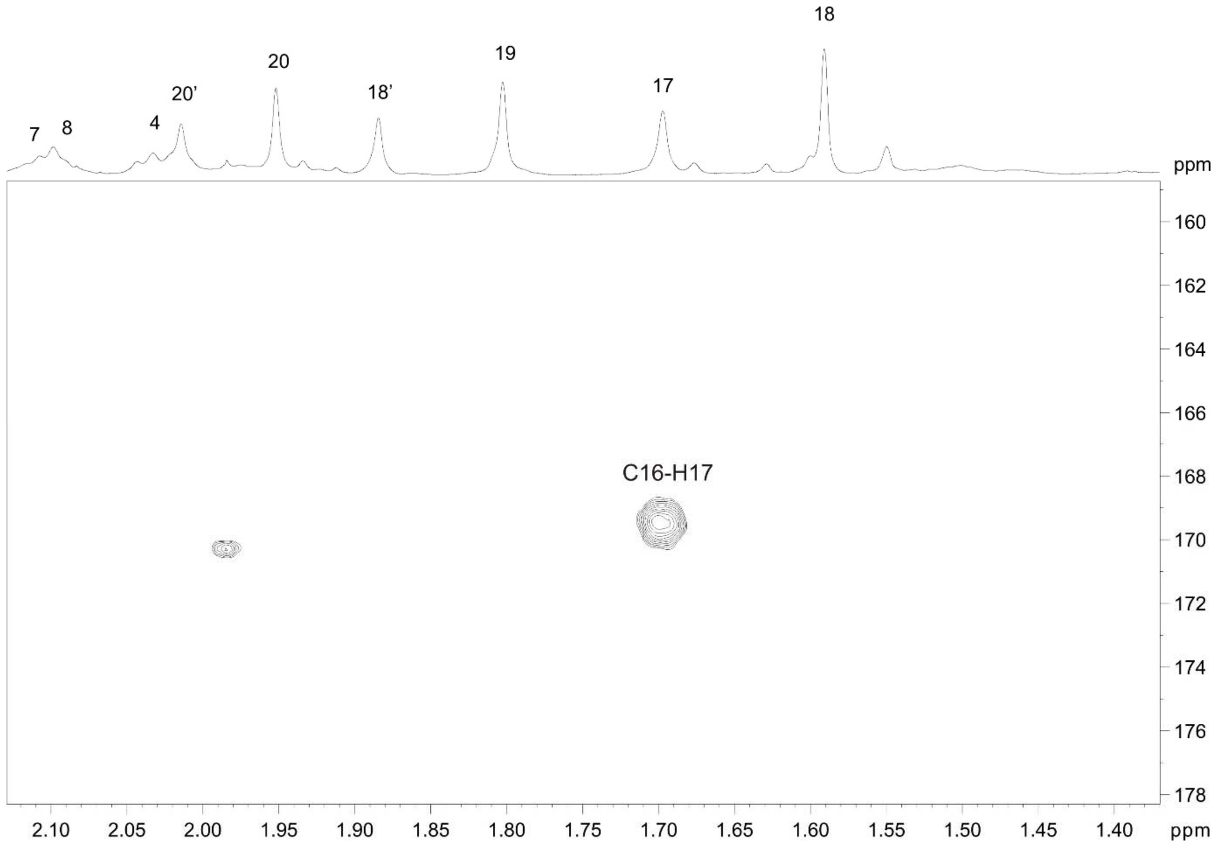

Figure S10: A detail of the HMBC spectrum of gemmatoxanthin (700.13 MHz for ^{1}H, 176.05 MHz for ^{13}C, DMSO-*d*$_6$, 303.1 K).

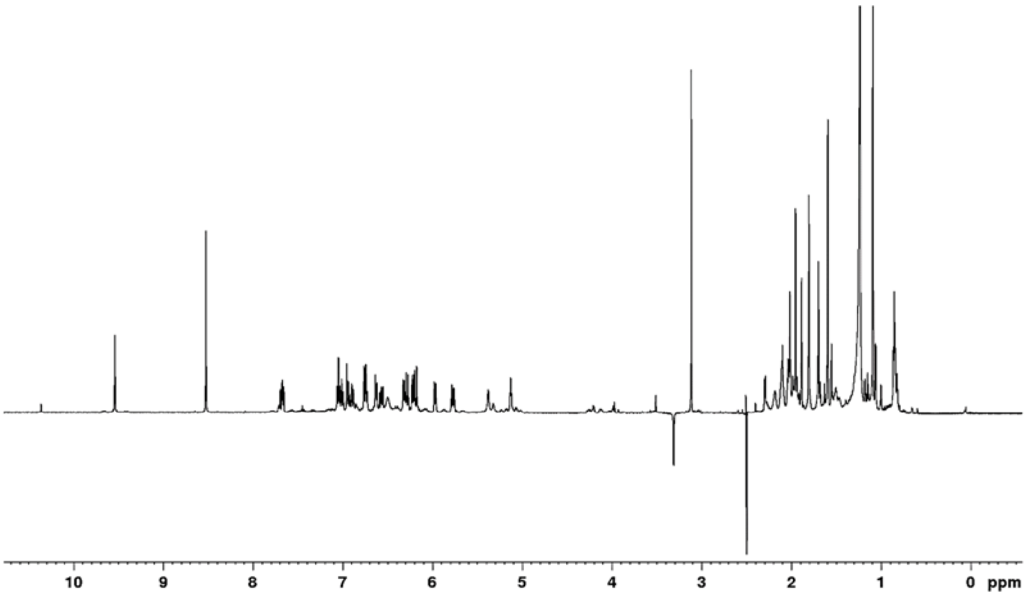

Figure S11: A ^{1}H NMR spectrum of gemmatoxanthin (700.13 MHz, DMSO-d_6, 303.1 K)

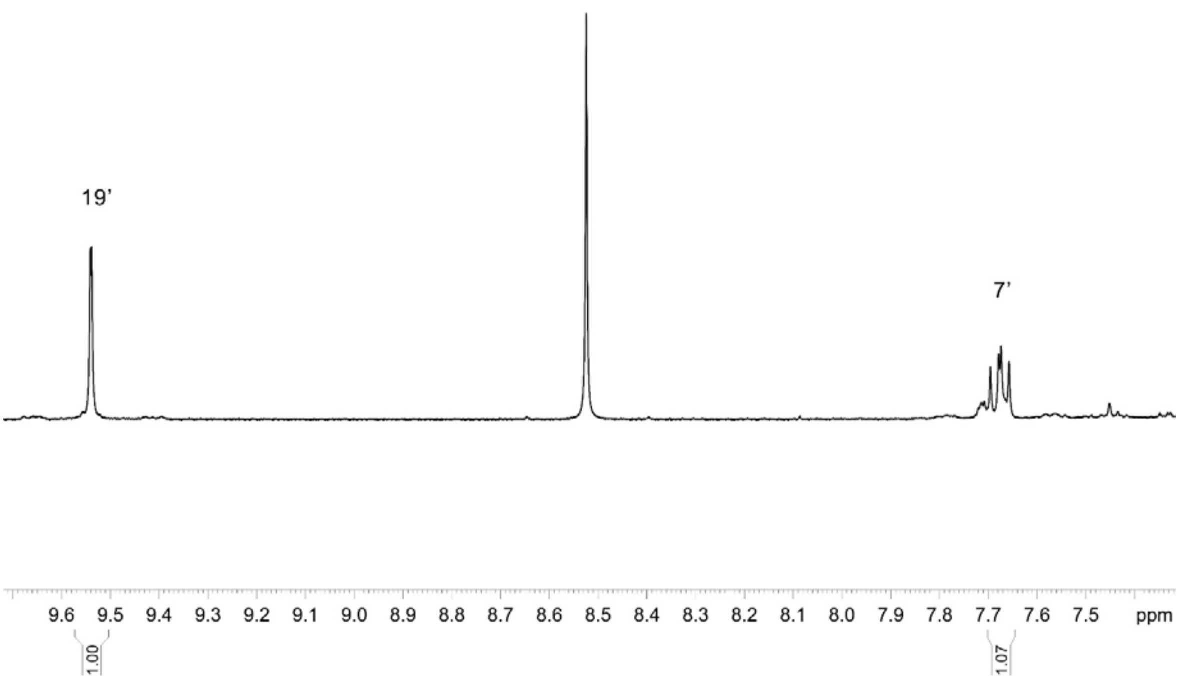

Figure S12: A detail of the ^{1}H NMR spectrum of gemmatoxanthin (700.13 MHz for ^{1}H, DMSO-d_6, 303.1 K).

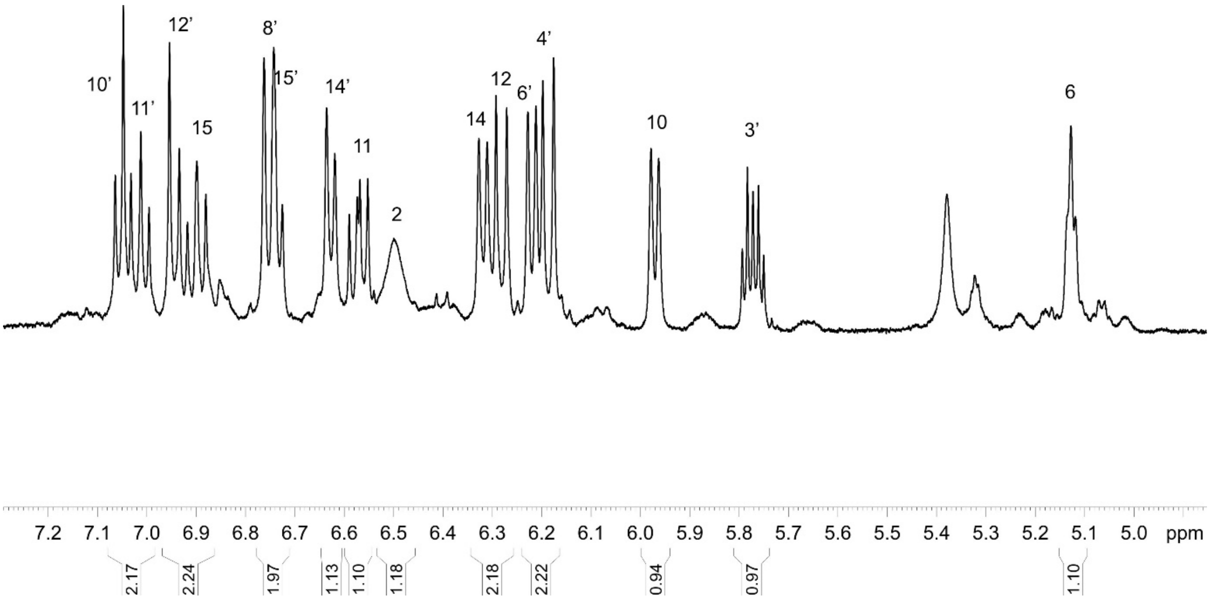

Figure S13: A detail of the ^{1}H NMR spectrum of gemmatoxanthin (700.13 MHz for ^{1}H, DMSO-d_6, 303.1 K).

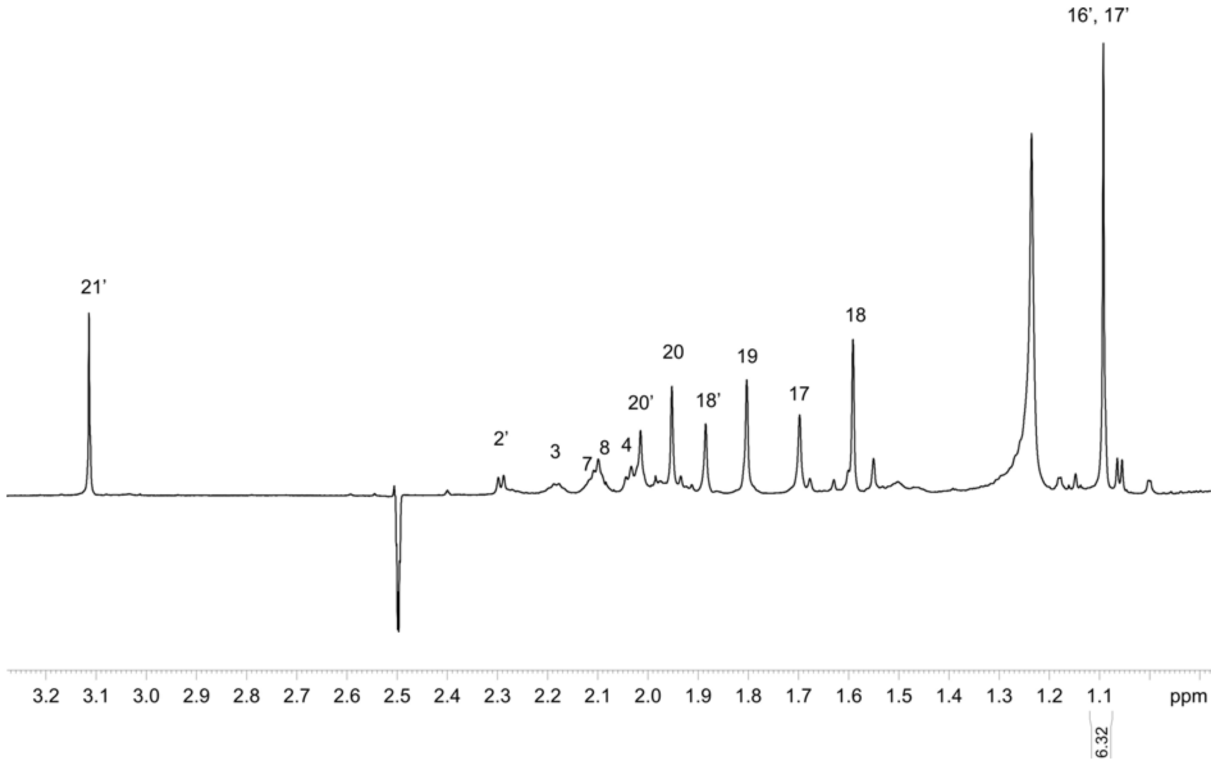

Figure S14: A detail of the ^{1}H NMR spectrum of gemmatoxanthin (700.13 MHz for ^{1}H, DMSO-d_6, 303.1 K) – the signals are not integrated because they are strongly effected by presaturation pulse.

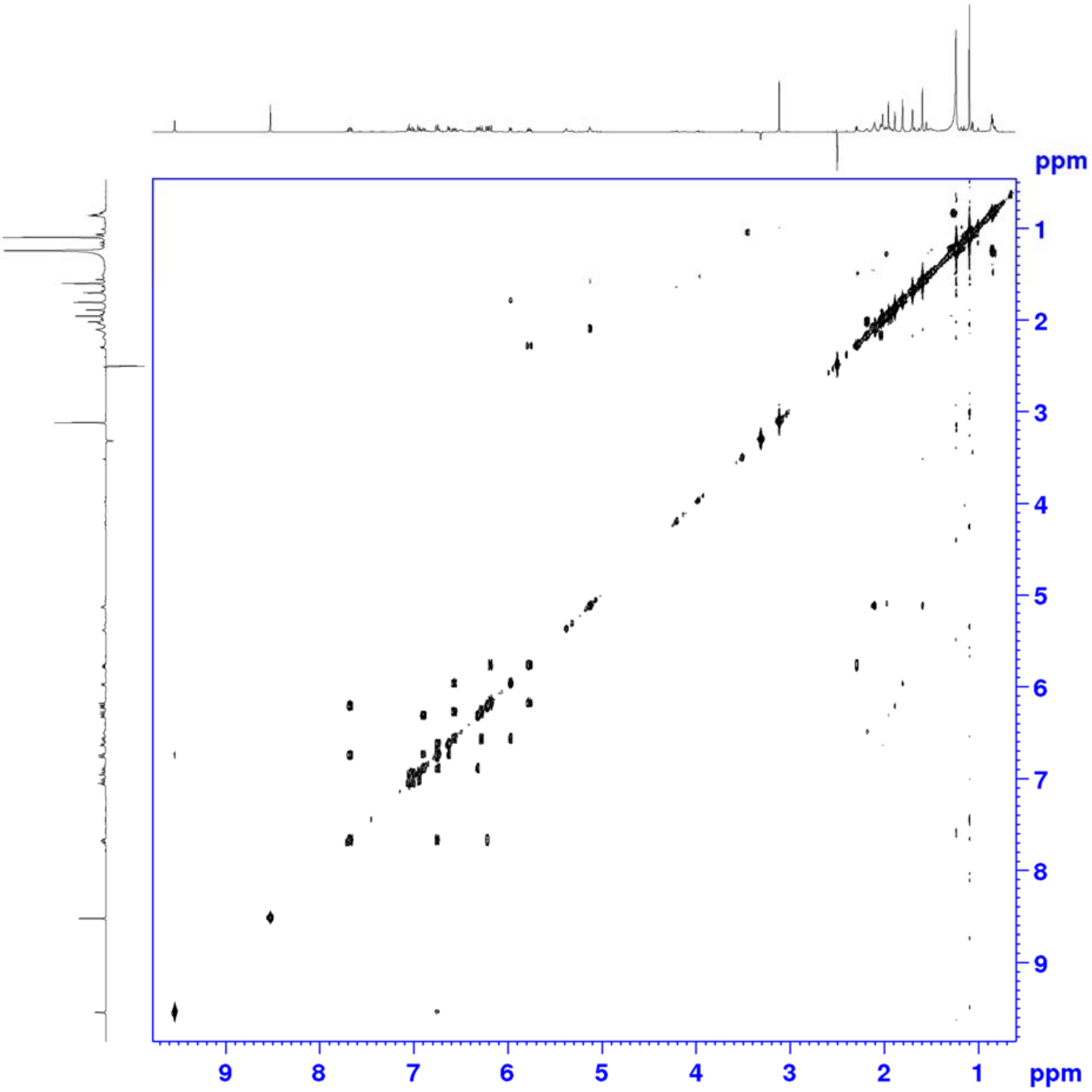

Figure S15: A COSY spectrum of gemmatoxanthin (700.13 MHz for ^{1}H, DMSO-d_6, 303.1 K)

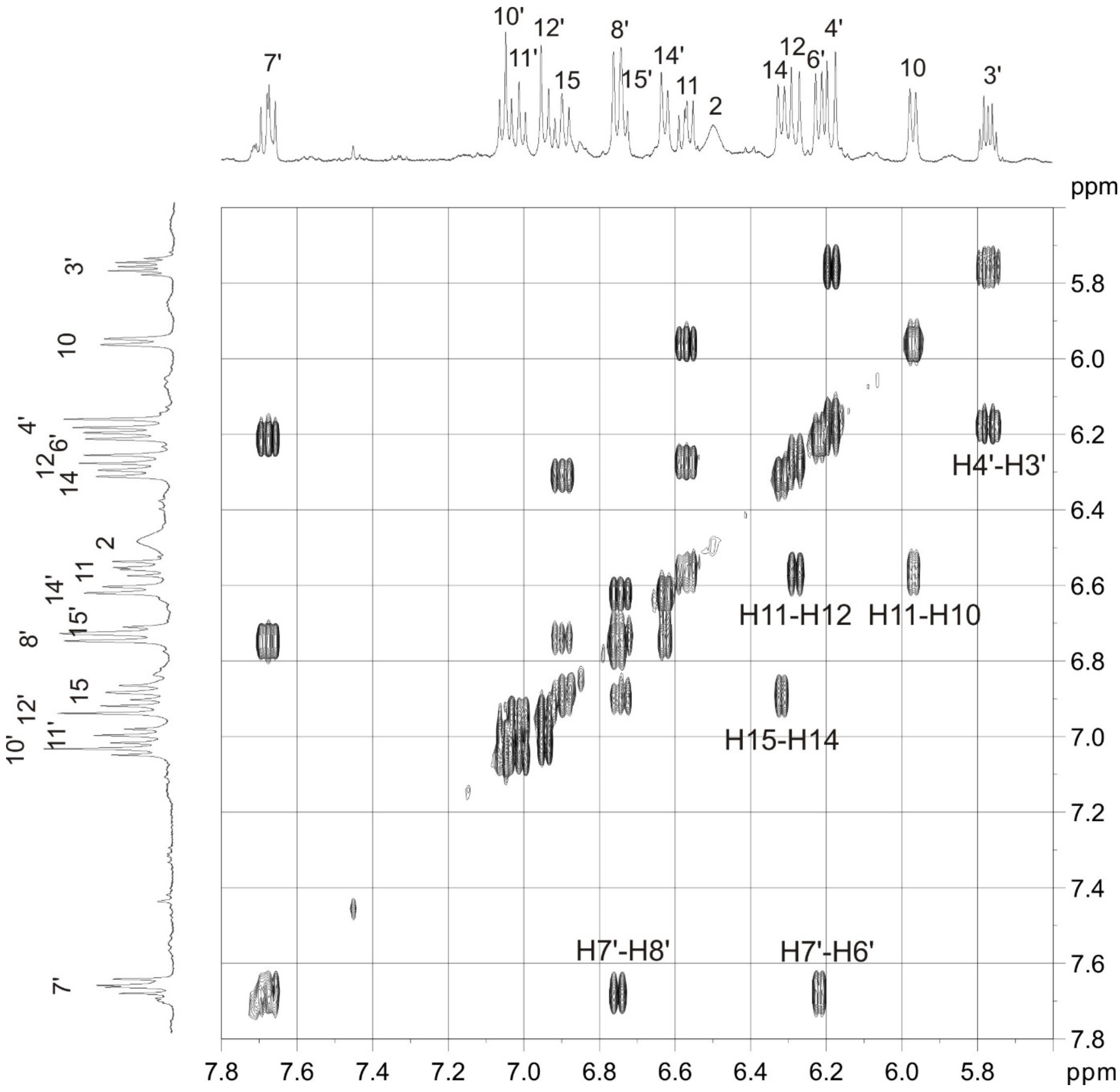

Figure S16: A detail of the COSY spectrum of gemmatoxanthin (700.13 MHz for ^{1}H, DMSO-d_6, 303.1 K).

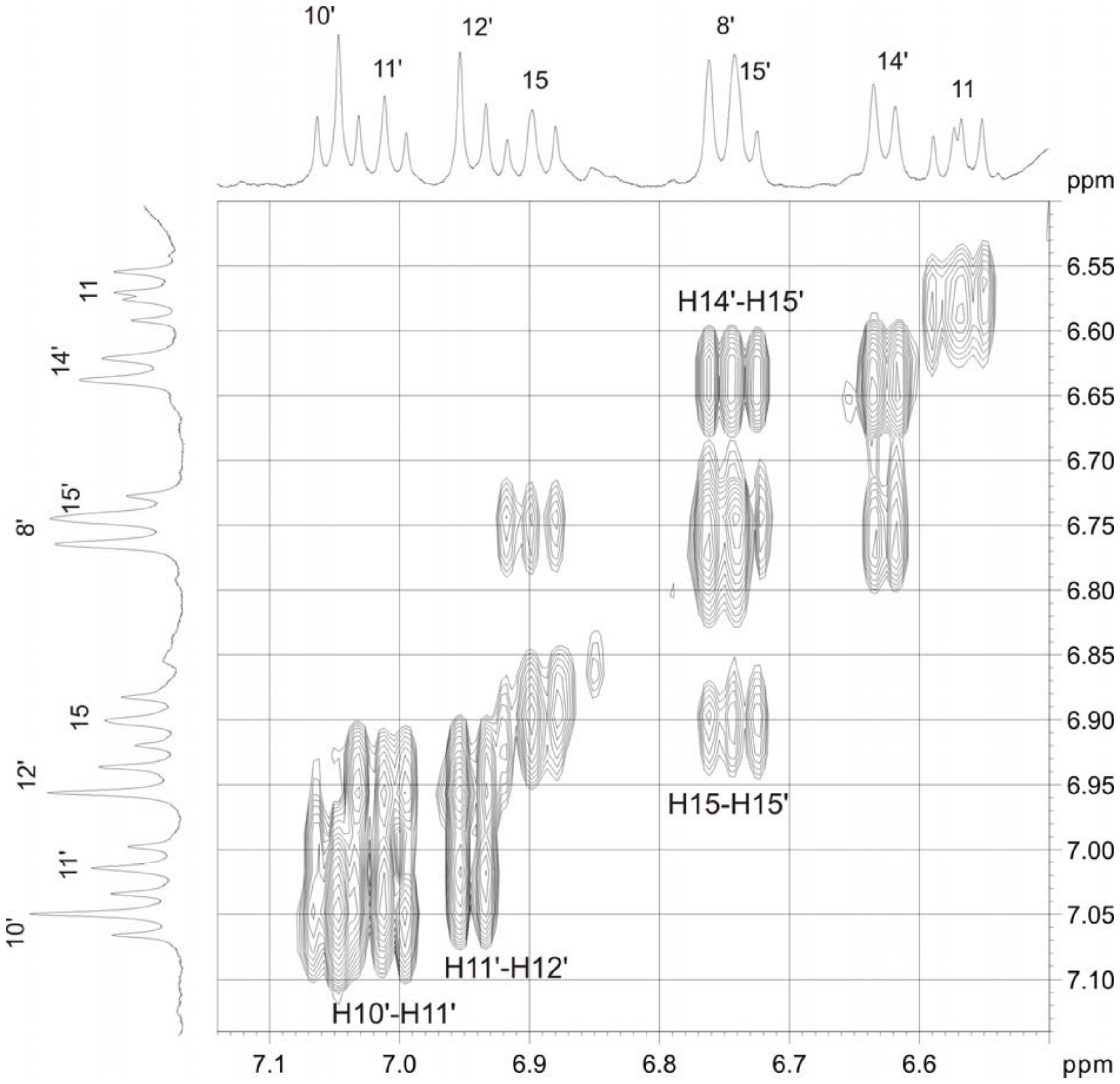

Figure S17: A detail of the COSY spectrum of gemmatoxanthin (700.13 MHz for ^{1}H, DMSO-d_6, 303.1 K).

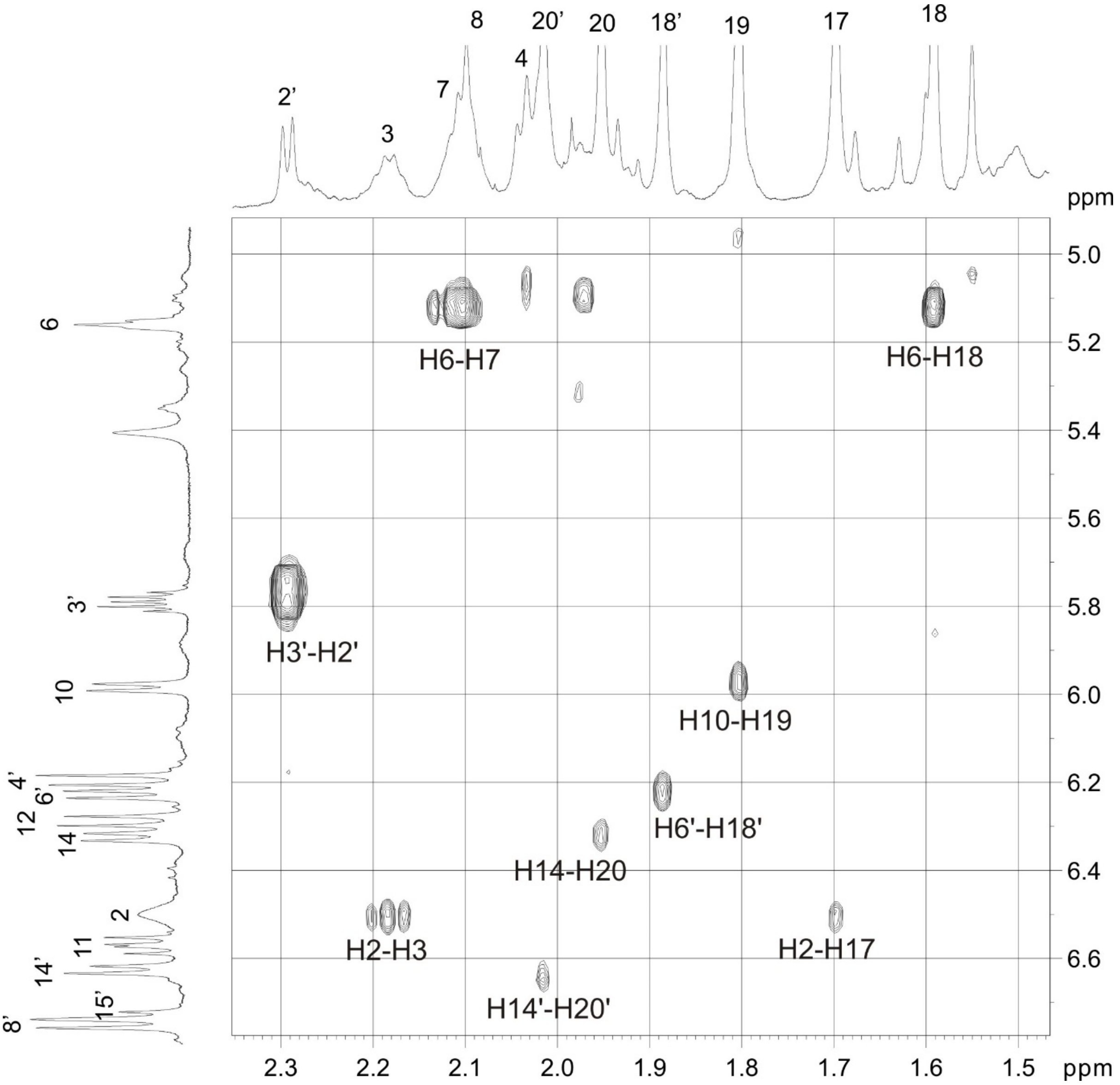

Figure S18: A detail of the COSY spectrum of gemmatoxanthin (700.13 MHz for ^{1}H, DMSO-d_6, 303.1 K).

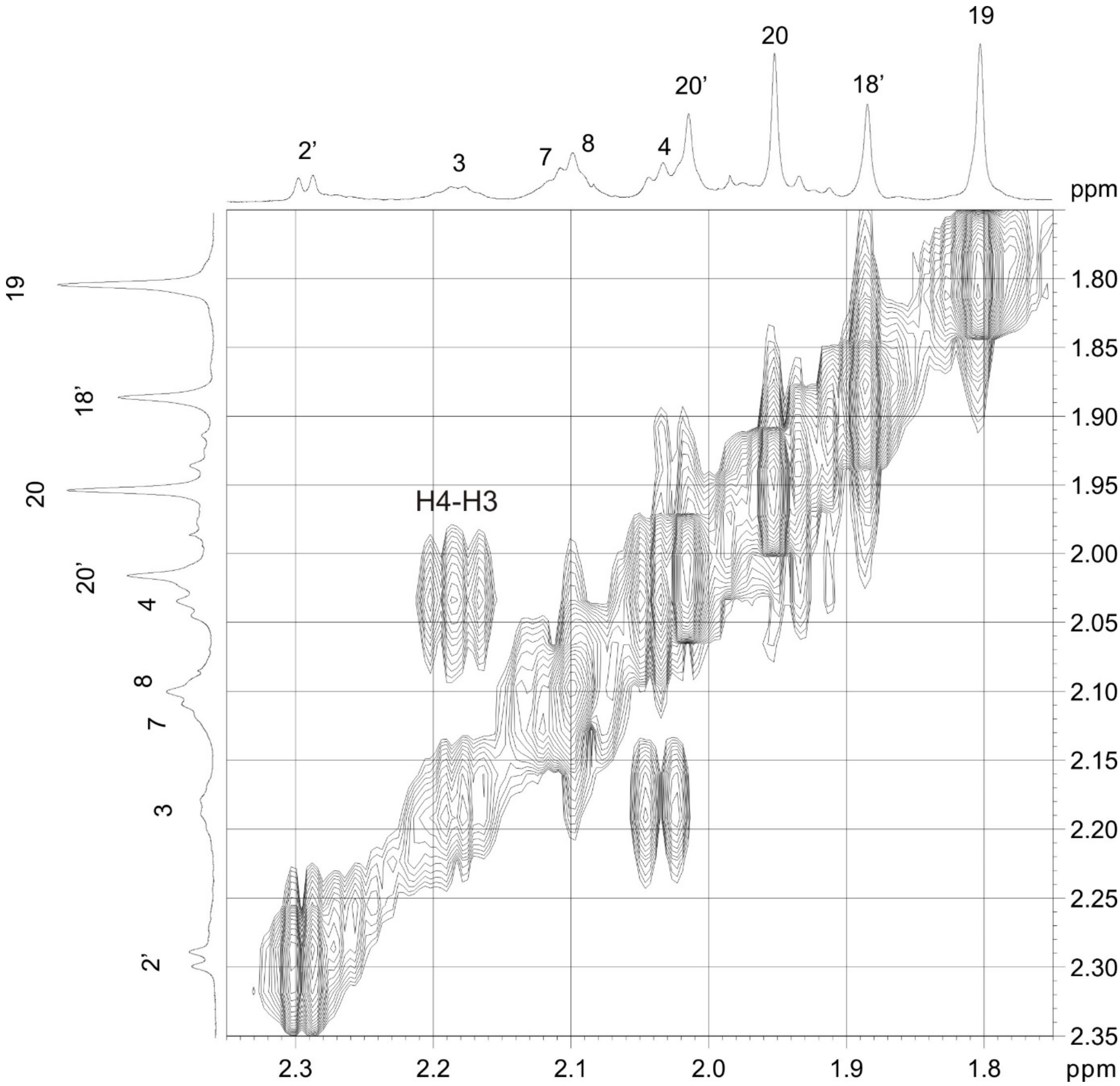

Figure S19: A detail of the COSY spectrum of gemmatoxanthin (700.13 MHz for ^{1}H, DMSO-d_6, 303.1 K).

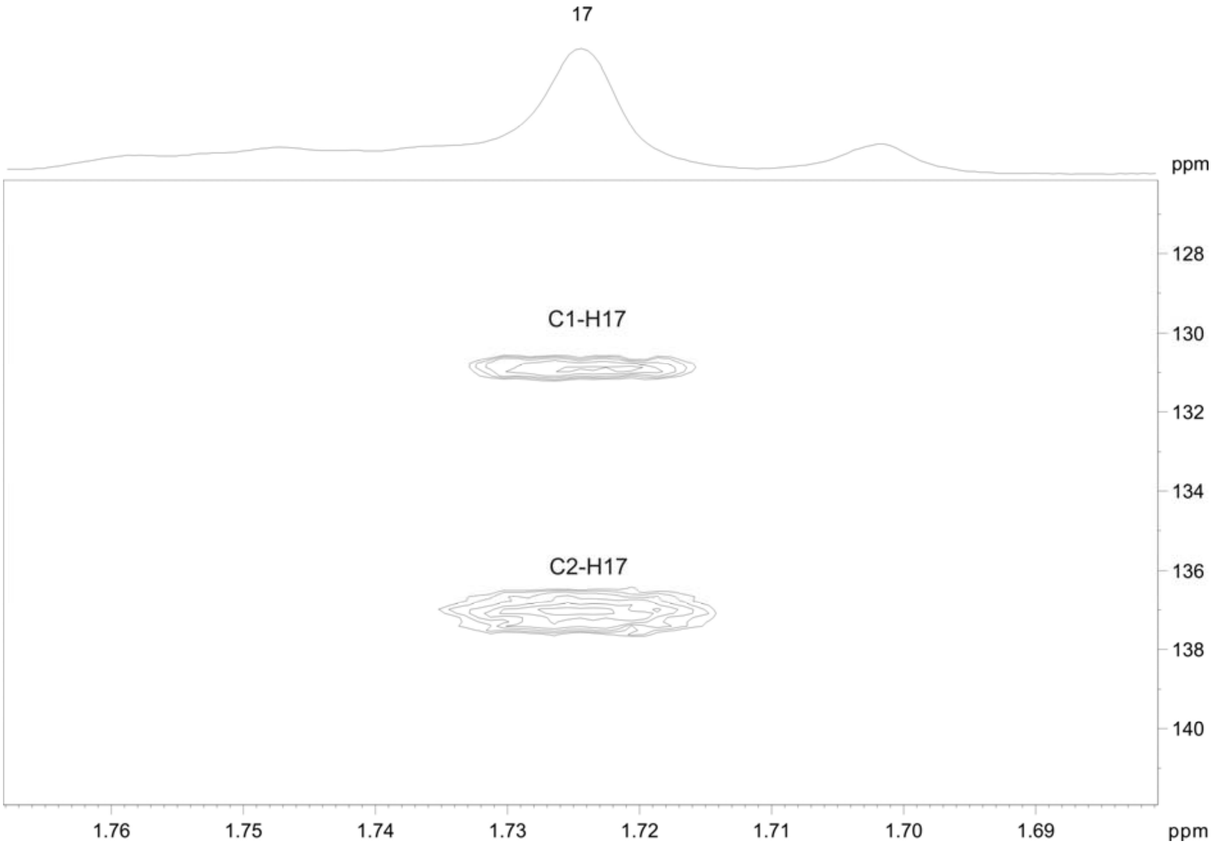

Figure S20: A detail of ^{1}H-^{13}C HMBC spectrum of gemmatoxanthin (700.13 MHz for ^{1}H, 176.05 MHz for ^{13}C, DMSO-d_6, 303.1 K) from the second batch of *G. phototrophica* cultivated in ^{13}C labelled SILEX *E. coli* media

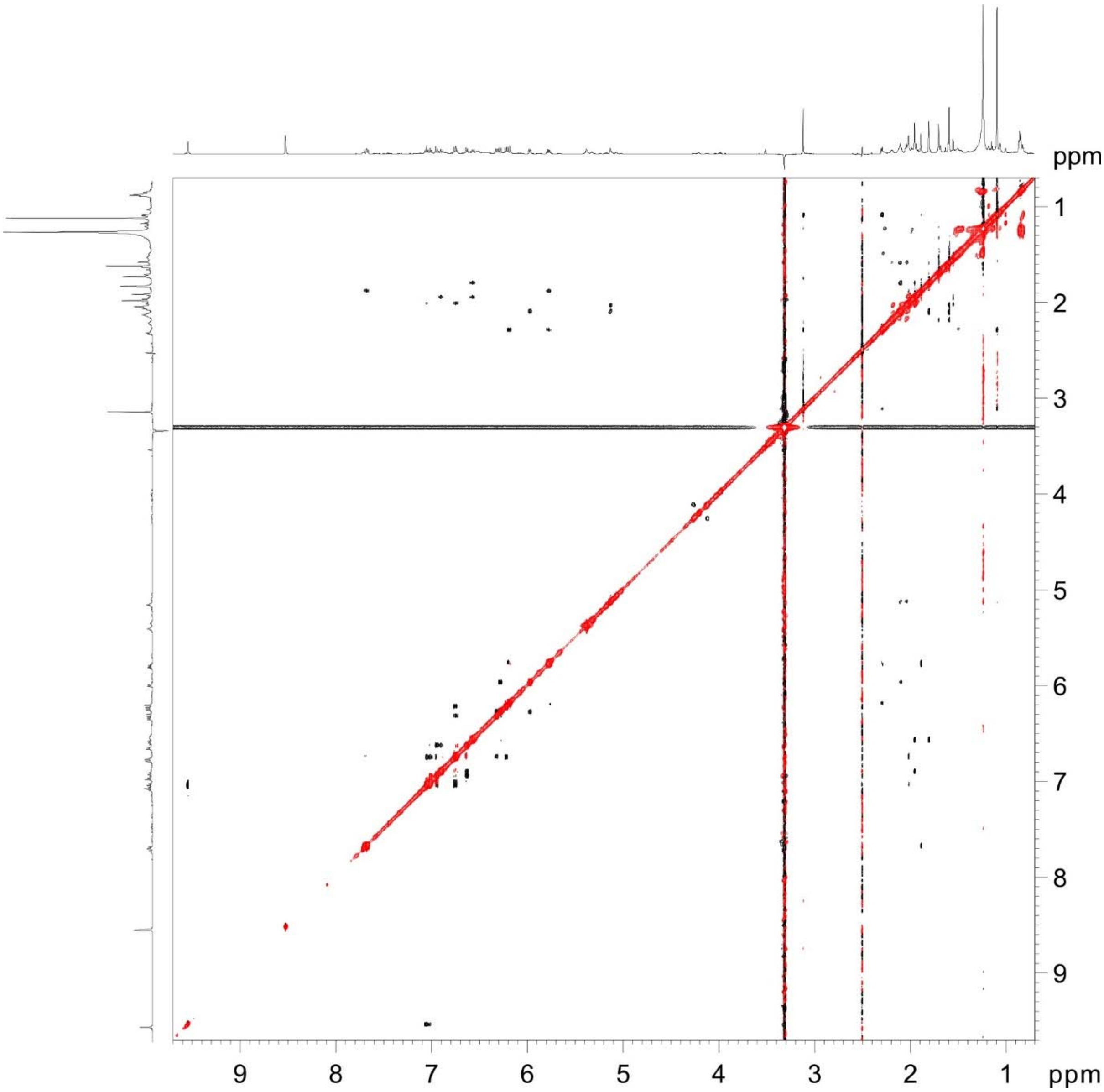

Figure S21: A detail of the ROESY spectrum of gemmatoxanthin (700.13 MHz for ^{1}H, DMSO-d_6, 303.1 K).

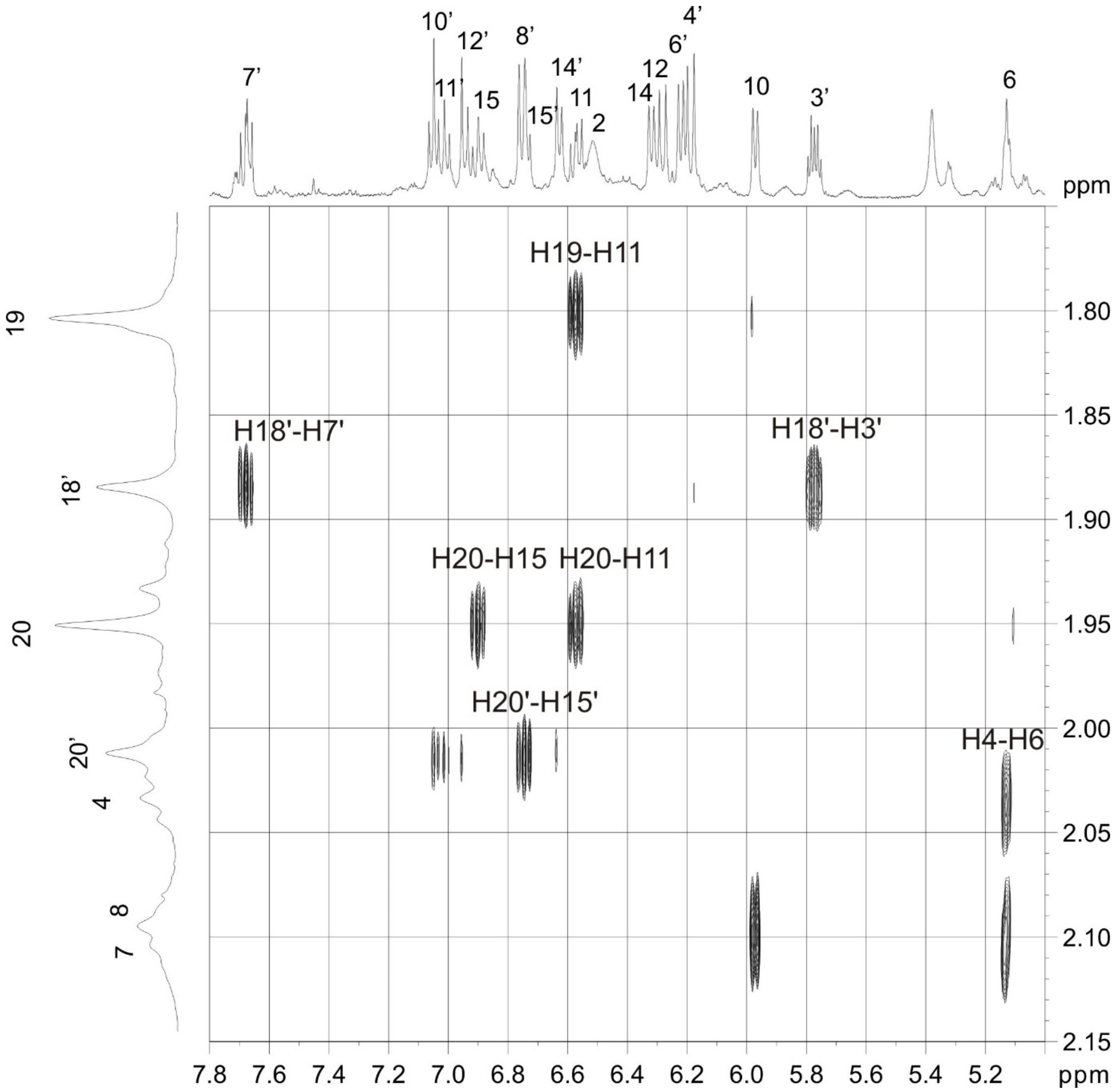

Figure S22: A detail of the ROESY spectrum of gemmatoxanthin (700.13 MHz for ^{1}H, DMSO-d_6, 303.1 K).

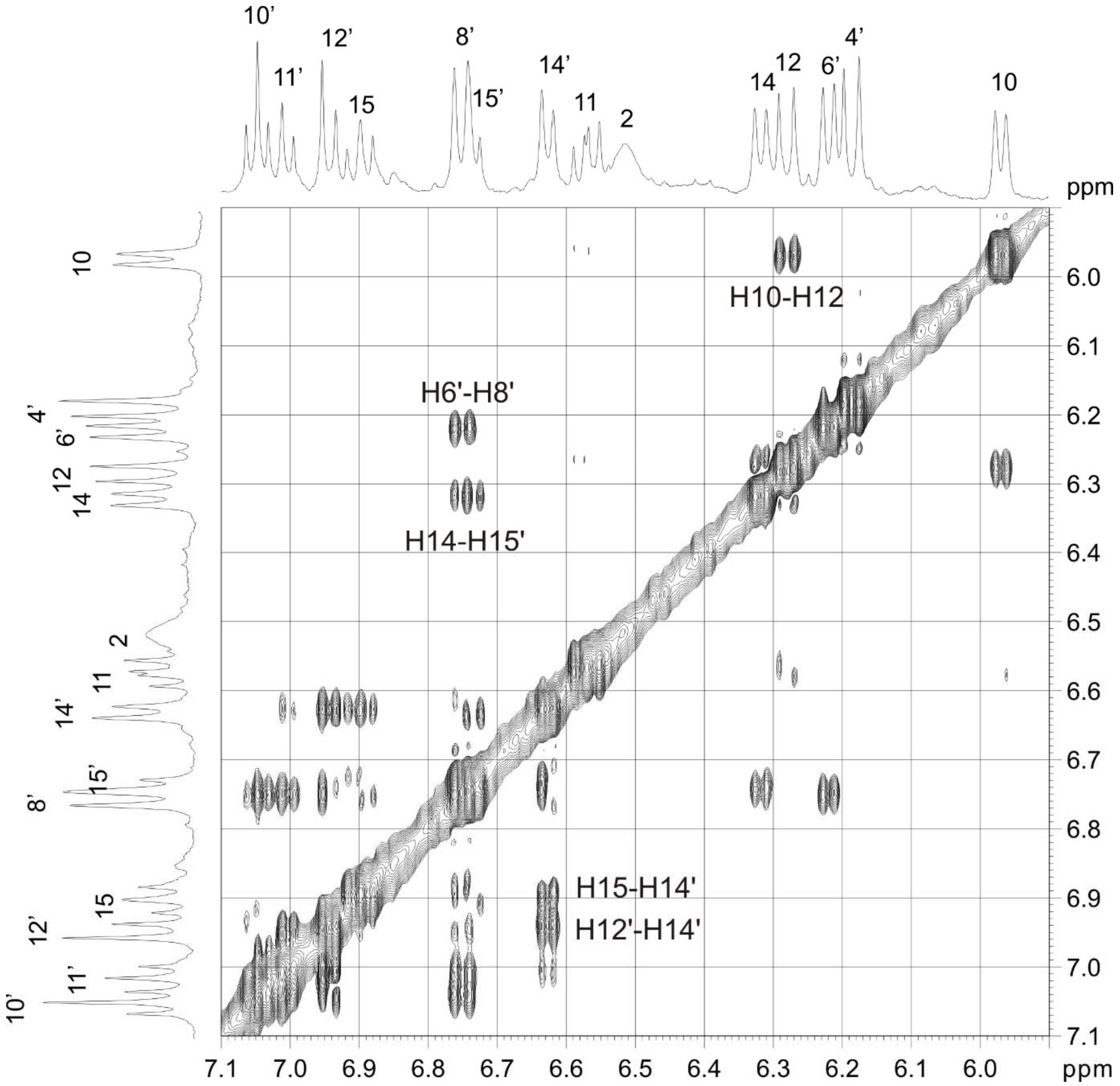

Figure S23: A detail of the ROESY spectrum of gemmatoxanthin (700.13 MHz for ^{1}H, DMSO-d_6, 303.1 K).

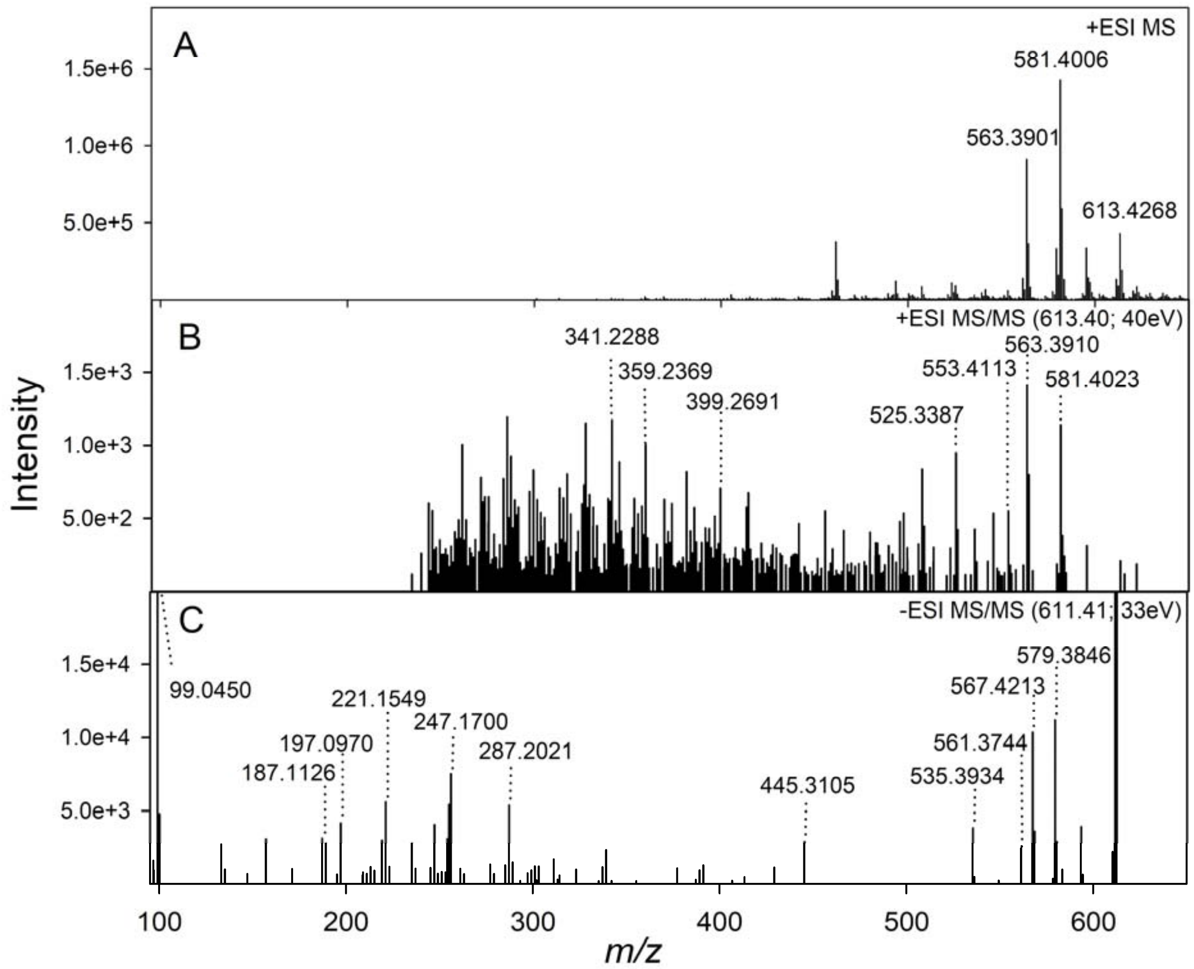

Figure S24: Fragmentation analysis of gemmatoxanthin. (A) MS spectrum of gemmatoxanthin in positive ESI ionization. (B) Fragmentation spectrum of gemmatoxanthin molecular ion (*m/z* 613.4) obtained in positive ionization mode. (C) Fragmentation spectrum of gemmatoxanthin molecular ion (*m/z* 611.41) obtained in negative ionization mode. Only the crucial fragments relevant for gemmatoxanthin characterization as depicted in Figure 3 are annotated.

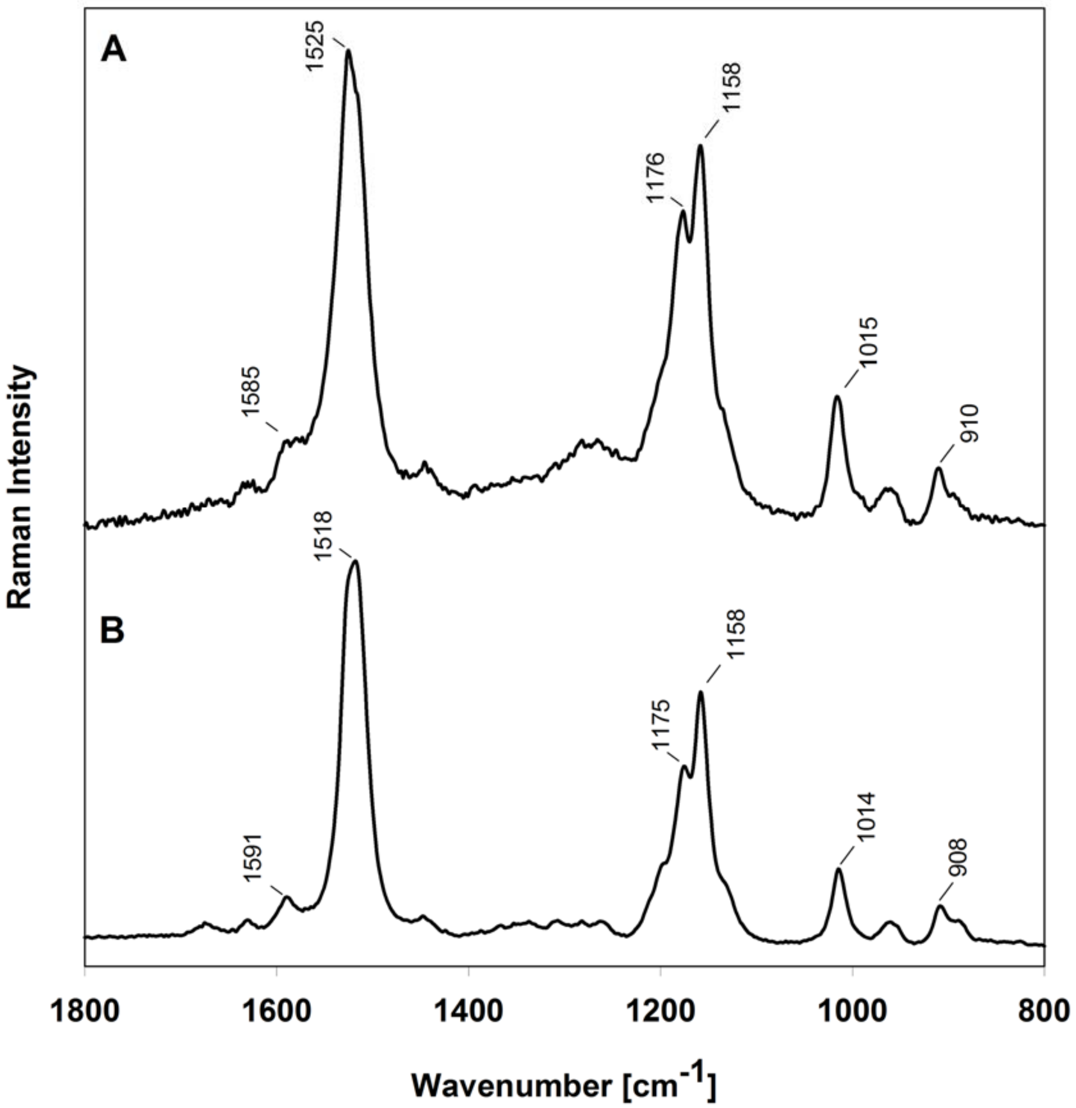

Figure S25: Raman spectra of gemmatoxanthin at (A) 785nm and (B) 532nm.
